# TrkA is amplified in malignant melanoma patients and induces an anti-proliferative response in cell lines

**DOI:** 10.1186/s12885-015-1791-y

**Published:** 2015-10-24

**Authors:** Luigi Pasini, Angela Re, Toma Tebaldi, Gianluca Ricci, Sebastiana Boi, Valentina Adami, Mattia Barbareschi, Alessandro Quattrone

**Affiliations:** 1 Centre for Integrative Biology (CIBIO), University of Trento, Trento, Italy; 2 Department of Pathology, Santa Chiara Hospital, Trento, Italy; 3 High Throughput Screening Facility, Centre for Integrative Biology (CIBIO), University of Trento, Trento, Italy

**Keywords:** TrkA (NTRK1), Genomic amplification, Malignant melanoma, Oncogene-induced growth arrest, p21^cip1^ (CDKN1A)

## Abstract

**Background:**

The nerve growth factor (NGF) receptor tyrosine-kinase TrkA is a well-known determinant of the melanocytic lineage, through modulation of the MAPK and AKT cascades. While TrkA gene is frequently rearranged in cancers, its involvement in malignant melanoma (MM) development is still unclear.

**Methods:**

We analyzed a dataset of primary cutaneous MM (*n* = 31) by array comparative genomic hybridization (aCGH), to identify genomic amplifications associated with tumor progression. The analysis was validated by genomic quantitative PCR (qPCR) on an extended set of cases (*n* = 64) and the results were correlated with the clinical outcome. To investigate TrkA molecular pathways and cellular function, we generated inducible activation of the NGF-TrkA signaling in human MM cell lines.

**Results:**

We identified amplification of 1q23.1, where the TrkA locus resides, as a candidate hotspot implicated in the progression of MM. Across 40 amplicons detected, segmental amplification of 1q23.1 showed the strongest association with tumor thickness. By validation of the analysis, TrkA gene amplification emerged as a frequent event in primary melanomas (50 % of patients), and correlated with worse clinical outcome. However, experiments in cell lines revealed that induction of the NGF-TrkA signaling produced a phenotype of dramatic suppression of cell proliferation through inhibition of cell division and pronounced intracellular vacuolization, in a way straightly dependent on NGF activation of TrkA. These events were triggered via MAPK activity but not via AKT, and involved p21^cip1^ protein increase, compatibly with a mechanism of oncogene-induced growth arrest.

**Conclusions:**

Taken together, our findings point to TrkA as a candidate oncogene in MM and support a model in which the NGF-TrkA-MAPK pathway may mediate a trade-off between neoplastic transformation and adaptive anti-proliferative response.

**Electronic supplementary material:**

The online version of this article (doi:10.1186/s12885-015-1791-y) contains supplementary material, which is available to authorized users.

## Background

The neurotrophic tyrosine kinase receptor type 1 (NTRK1) or TRK1-transforming tyrosine kinase protein (TrkA) is encoded in humans by the *NTRK1* gene, located in the chromosome region 1q23.1. TrkA specifically mediates the multiple effects of the nerve growth factor (NGF) signaling through receptor autophosphorylation and downstream induction of the mitogen-activated protein kinase (MAPK) and protein kinase B (PKB/AKT) pathways [1]. Although ubiquitously expressed, TrkA is pivotal in mediating survival and differentiation of neuroectoderm-derived cells, as neurons and melanocytes [2]. During both development and adult life, overall levels of NGF determine a balance between cell proliferation and apoptosis of target cells [3]. These effects are usually modulated by the p75 neurotrophin receptor (p75NTR), an accessory receptor of TrkA that, by communicating through convergence of signal transduction, can increase the response to NGF or can signal by its own alternative function [3]. Given the complexity of this signaling and the dual biological role of the NGF-TrkA axis in modulating either pro-survival or pro-apoptotic responses, regulation of malignant transformation by the NGF pathway is not completely understood. To date, TrkA signaling has been intensively dissected for tumors of the neuroectodermal lineage like neuroblastomas where, although TrkA is overexpressed through genomic rearrangements and can contribute to tumor onset, it seems to have a protective effect against later unfavorable outcome [4]. However, probably as a consequence of its predominant function in stimulating cell proliferation, deregulation of the TrkA pathway is common in cancers [5]. In this context, chromosomal translocation of region 1q23.1 is known as the major mechanism in oncogenic activation of TrkA, being observed in several cancer types [6].

The fact that NGF and other neurotrophins are required for regulating melanocyte fate [7] underlines the importance of Trk family members in the skin [8] and poses the basis for investigating their activity in malignancy onset and progression. However, very little is known about the molecular function of Trk receptors in melanocyte biology, and the exact mechanisms by which the NGF-TrkA signaling may act in melanocytic disorders remain largely unknown. Cutaneous malignant melanoma (MM) is a deadly cancer of melanocyte origin, for which conventional therapies become ineffective once the tumor metastasizes [9]. In particular, a large proportion of primary MMs harbors alterations in the BRAF kinase that lead to the constitutive activation of the MAPK pathway [10]. But, despite its aggressive behavior, MM is a typical example of tumor where hyperactivation of MAPK signaling may induce a strong negative feedback, resulting in reduction of the mitogenic stimulus [11]. This mechanism is evident in benign nevi, where a growth arrest program is operated by oncogenic BRAF [12]. The natural propensity of melanocytic cells to elicit a physiological protective response against neoplastic progression is exploited as a key factor for clinical treatment of MM [13]. Hence, the identification of pathways that regulate melanomagenesis should serve for the development of novel therapeutic modalities.

Recent advancements in microarray technologies have revealed the complexity of genomic rearrangements occurring in MM [14], with profound patterns of copy number alterations (CNAs) that can arise already at its early stages [15]. However, the discovery of specific driver genes and the accurate profiling of genomic mutations and CNAs in MM have been mainly based on MM cell lines derived from metastatic samples [16, 17] or have included a restricted cohort of clinical primary tumors [18], limiting the detection of novel candidate alterations that may originate in the primary MM.

Although oncogenic activation of TrkA through kinase-domain fusion has been recently observed in spitzoid melanoma-like lesions [19] and region 1q23.1 is gained or amplified in a variety of other cancers [20, 21], acquisition of TrkA genomic amplification in MM has never been reported. In this study, we identify amplification of TrkA as a candidate locus for melanomagenesis in a subset of primary MM clinical samples, previously analyzed by array comparative genomic hybridization (aCGH) [15]. Subsequent experiments in MM cell lines upon conditional activation of NGF-TrkA signaling reveal that, while TrkA is amplified in MM and may act as an oncogene via the AKT pathway, it can also mediate an oncogene-induced type of proliferation arrest via MAPK activity and p21^cip1^ induction. This effect may suggest a role of TrkA in coupling with the MAPK pathway to promote susceptibility of MM cells to physiological anti-oncogenic protection.

## Methods

### Tumor specimens from patients

We collected 64 formalin-fixed and paraffin-embedded (FFPE) samples of primary cutaneous MM from the Surgical Pathology Unit of the S. Chiara Hospital in Trento, Italy. The study was approved by the Research Ethics Committee for Clinical Experimentation of the Trentino Public Healthcare Agency, Italy, and each patient signed formal written informed consent for sampling and research. Samples were diagnosed by expert pathologists (SB and MB), according to the classification system of the American Joint Committee on Cancer [22]. Clinical features of the primary MMs and patients’ follow-up data are summarized in Additional file 1: Table S1. The sample ID indicated in any of the tables cannot be linked back to any of the patients.

### Cell lines

MM cell lines SK-MEL-28 and G-361 were a gift of Alberto Inga (CIBIO, University of Trento, Italy) and were originally obtained from the ICLC Interlab Cell Line Collection (Istituto Nazionale per la Ricerca sul Cancro, Genova, Italy). SK-MEL-28 cells were grown in DMEM supplemented with 10 % fetal bovine serum (FBS), 2 mM L-Glutamine, 1 % non-essential amino acids, penicillin, and streptomycin. G-361 cells were cultured in EMEM, supplemented with 10 % FBS, 2 mM L-Glutamine, 1 % non-essential amino acids, penicillin, and streptomycin. SK-MEL-28-TrkA and G-361-TrkA or SK-MEL-28-E and G-361-E, were obtained by lentiviral infection with TrkA-containing plasmid or empty vector, respectively, and were maintained in the same culture medium as the original stock.

### Genome profiling of clinical samples

Genomic copy number aCGH profiles of 31 MM samples, available as series GSE45354, at Gene Expression Omnibus (GEO) repository (http://www.ncbi.nlm.nih.gov/geo/), were analyzed as previously described [15]. In brief, the array CGH was performed using the Agilent 8x60K human CGH oligo microarray chip (Agilent Technologies, Santa Clara, CA; 021924 SurePrint G3 Human CGH 8x60K Microarray, cat. G4450A), mapped to the human genome (USCS genome browser Human, Feb. 2009, GRCh37/hg19). The scanned microarray TIFF images were acquired with the Agilent DNA Microarray Scanner G2505C, by the manufacturer’s software (Agilent ScanControl 8.1.3), and analyzed using the Agilent Feature Extraction Software version 10.7.7.1. The analysis of raw aCGH data was then conducted via the R environment for statistical computing (http://www.r-project.org/) using packages provided by the Bioconductor library (http://www.bioconductor.org/). Hotspots of minimal common regions of amplification were defined as the minimal regions of overlap shared by at least three samples with a maximum length of 2.5 Mb [20].

### DNA extraction from clinical samples and genomic real-time quantitative PCR

Genomic DNA (gDNA) was isolated from all FFPE archival samples using an optimized DNA isolation protocol based on a Qiagen extraction kit (#51306; Qiagen), as previously detailed [15]. Quantitative PCR (qPCR) validation of genomic copy number was performed by using the laminin alpha 1 (*LAMA1*) gene, located in 18p11.31, as reference gene, since this locus showed absence of CNAs in 97 % of cases from our aCGH dataset. As diploid calibrator, a pooled FFPE gDNA of 10 healthy patients with inflammation of the vermiform appendix was used. Two benign nevi were used as an additional diploid control. The reaction was performed by using the commercially available FAM-labeled TaqMan Copy Number Assay (Life Technology) for *LAMA1* exon 3 (Hs00282410_cn), *CDKN2A* exon 5 (Hs03714372_cn), and *NTRK1* intron 3 (Hs05769842_cn). A 10 μl reaction was prepared with 5 μl of KAPA PROBE-FAST qPCR Master Mix (2X) ABI Prims (Kapabiosystems), 0.5 μl of TaqMan assay (20X), and 10 ng of template gDNA. Thermal cycling conditions consisted of an initial cycle at 95 °C for 10 min, followed by 40 cycles each of 15 s 95 °C and 1 min 60 °C. Comparative cycle threshold (Ct) values for each target gene were calculated by Bio-Rad CFX Manager 2.1 software (Bio-Rad Laboratories Inc.) using regression mode and relative copy number ratio was measured by the *E* ^*ΔCt*^ method over the reference gene *LAMA1*, where *E* is the PCR efficiency calculated by standard curves generated from dilution series of calibrator gDNA, as previously described [15]. Experiments were repeated in two independent replicates, where PCR for each assay was performed in three internal replicates. Diploid copy number was set as a fold change of 1; gain of one extra genomic copy was defined when fold change over diploid calibrator was between 1.25 and 1.75; amplification was defined as an increase in fold change above 1.75; hemizygous deletion was determined as a fold change between 0.75 and 0.5; homozygous deletion was defined as fold-change decrease below 0.5 [15, 23].

### Quantitation of DNA copy number and mRNA expression for cell lines

Total gDNA from MM cells was extracted with DNeasy Blood & Tissue kit (Qiagen). Genomic copy number of TrkA and CDKN2A was quantified by comparison with gDNA of normal melanocytes (#C-024-5C; HEMaLP, Life Technology), using the same primer set and protocols as previously described for tissue samples. Relative copy number ratio was measured by applying regression mode, as calculated by the Bio-Rad CFX Manager 2.1 software, and the *ΔΔCt* method Ct for normalization of Ct values to *LAMA1* as internal reference gene [24]. The experiment was repeated twice.

Total RNA from MM cells was extracted by using RNeasy Plus mini Kits (Qiagen) and reverse-transcribed using iScript^TM^ cDNA Synthesis Kit (Bio-Rad). The obtained cDNA was subjected to real-time qPCR by TaqManGene Expression Assay (Life Technologies). Commercially available FAM-labeled TaqMan assays were used for *LAMA1* (Hs00300550_m1) and *NTRK1* (Hs01021011_m1). A 10 μl reaction was prepared with 5 μl of KAPA PROBEFAST qPCR Master Mix (2X) ABI Prims (#KK4702; Kapabiosystems, Woburn, MA), 0.5 μl of TaqMan assay (20X), 100 ng of template cDNA, and run on Bio-Rad CFX384 Real-Time PCR Detection System (Bio-Rad). PCR cycles were: 95 °C for 3 min, followed by 40 cycles at 95 °C for 10 s and 60 °C for 30 s. Values of Ct were calculated by Bio-Rad CFX Manager 2.1 software, using regression mode, and *ΔΔCt* method was used for expression quantification using the Ct of *LAMA1* for normalization [24]. Results were obtained as a mean of three experiments.

### Vectors and lentiviral transduction

The human TrkA gene (splice variant NTRK1-001, RefSeq NM_001012331.1) was subcloned from the original pCMV5-TrkA (Addgene Plasmid 15002; ref. [25]) into SalI-XbaI sites of the doxycycline-inducible Tet-On lentiviral vector pLenti-CMV/TO-eGFP-Puro (Addgene Plasmid 17481; ref. [26]), by replacing the eGFP sequence, and the construct was verified by Sanger sequencing. MM cells SK-MEL-28 and G-361 were transduced with the tetracycline-repressor expression vector pLenti-CMV-TetR-Blast (Addgene Plasmid 17492; ref. [26]) before transduction with pLenti-CMV/TO-TrkA-Puro or the pLenti-CMV/TO-Puro empty vector. Lentiviral particles were produced by co-transfecting the transfer plasmids with packaging vector pCMV delta R8.2 (Addgene plasmid 12263; Didier Trono) and envelop plasmid pMD2.G (Addgene plasmid 12259; Didier Trono) into HEK-293-T cells (ICLC Interlab Cell Line Collection), in a penicillin/streptomycin-free Opti-MEM® culture medium (Life Technology), with 0.5 mg/ml Polyethylenimine (Sigma-Aldrich), based on Trono lab protocols (http://tronolab.epfl.ch). Viral titer in the supernatant was established at 0.5 transducing units (TU) per reaction, as measured by SYBR Green I-based PCR-enhanced reverse transcriptase (SG-PERT) assay [27]. Parallel infection efficiency of pLenti-CMV/TO-eGFP-Puro control plasmid was above 60 % at 96 h post infection, as quantified by the GFP signal. Transduced cells were selected for 6 days with puromycin 3 μg/ml (Sigma-Aldrich), starting at 48 h post-infection.

### Cell treatments

Before performing the experiments, transduced cells were allowed to adhere to the plate by growing for 16 h in complete melanoma cell medium. Afterwards, to induce TrkA expression cells were pre-treated with 500 ng/ml doxycycline (Sigma-Aldrich) for 48 h, either in medium 2 % FBS or FBS-free medium, and doxycycline was maintained during the entire course of the experiments. To test the activation of NGF-TrkA downstream pathways, cells were treated with 100 ng/ml β-NGF (#PHG0126; Life Technology) for 15 min in FBS-free medium. A dose–response curve was measured by incubating the cells for 15 min in FBS-free medium with 6.25, 12.5, 25, 50, and 100 ng/ml β-NGF. To activate NGF-TrkA signaling before phenotypic assays, cells were treated with 100 ng/ml β-NGF for 24 h or 48 h. To specifically block the MAPK pathway, cells were incubated with 5 μM U0126 (Promega) in the presence or absence of NGF. To inhibit the AKT pathway, cells were incubated with 25 μM LY294002 (Promega) in the presence or absence of NGF. CEP-701 (Sigma-Aldrich) was used at 10 μM, as a broad inhibitor of kinase signaling. Control experiments were conducted in the absence of doxycycline in 2 % FBS medium or FBS-free medium plus vehicle (DMSO). During treatment experiments, vehicle was either water (for NGF controls) or DMSO (for kinase inhibitor controls).

### Western blot analysis

Cells (approximately 0.5 x 10^6^) were harvested on ice in lysis buffer (50 mMTris-HCL pH 8, 150 mM NaCl, 1 % NP-40, 0.5 % sodium deoxycholate, 0.1 % SDS) supplemented with 1 μg/ml Pepstatina A (Sigma-Aldrich), protease inhibitor cocktail (Sigma-Aldrich) and phosphatase inhibitor cocktails 1/2 (Sigma-Aldrich). After determination of total protein content by the Bradford reagent (Sigma-Aldrich), 30 μg of protein extracts were resolved by SDS-PAGE gels and then blotted onto 0.2 μm nitrocellulose membrane (Bio-Rad). Unspecific protein binding was blocked by incubation for 1 h in 5 % Blotto non-fat dry milk (Santa Cruz Biotechnologies Inc.) in 0.1 % TBS-tween and membranes were incubated overnight at 4 °C with primary antibodies: rabbit anti-TrkA, 1:1000 (#06-574; Upstate); rabbit anti-phospho (Try490)-TrkA, 1:1000 (#9141S; Cell Signaling Technology Inc.); rabbit anti-ERK1/2, 1:2000 (sc-153; Santa Cruz); rabbit anti-phospho-ERK1/2, 1:1000 (#4370S; Cell Signaling); rabbit anti-AKT(pan), 1:1000 (#4691S; Cell Signaling); rabbit anti-phospho-AKT1, 1:1000 (Ab66138; abcam); mouse anti-p21^cip1^ (sc-397; Santa Cruz, 1:2000); mouse anti-eIF4E, 1:1000 (SC9976; Santa Cruz); mouse anti-p53, 1:5000 (sc-377567; Santa Cruz); mouse anti-Cyclin D1, 1: 1000 (ab101430; Abcam); mouse anti-β-tubulin (sc-53140; Santa Cruz, 1:5000); mouse anti-α-actinin (sc-17829; Santa Cruz, 1:6000); mouse anti-GAPDH (sc-32233; Santa Cruz; 1:5000). After washing, membranes were incubated for 1 h at room temperature, with goat anti-rabbit (sc-2004; Santa Cruz) or goat anti-mouse (sc-2005; Santa Cruz) secondary HRP-conjugated antibodies, diluted 1:10000 in blocking solution. Membranes were then washed and developed by using the ECL detection assay (Amersham Biosciences). After detection of phospho-TrkA, phospho-AKT, and phospho-ERK signals, the membranes were stripped with Re-Blot Plus Mild Solution (Merck Millipore) and re-blotted for total protein staining. Protein expression was quantified from digital images by Image Lab software (Bio-Rad), setting the global subtraction method for background. TrkA proteins typically correspond to two WB bands: the mature cell surface 140-kDa form and the immature 110-kDa form, which is subsequently modified by glycosylation in the ER before translocation to the membrane [28].

### Cell-cycle analysis

Cells were seeded (0.4 × 10^5^ cells/well) in a 6-well plate and allowed to adhere for 16 h in complete medium. After treatment, cells were centrifuged and processed with the Cycletest™ Plus DNA Reagent Kit (BD Biosciences) and incubated in Propidium Iodide (PI) labeling solution, following the manufacturer’s indications. Cell cycle analysis, by measuring DNA content, was performed by flow cytometry using a FACS Canto II instrument (BD Biosciences). FACSDiva™ Software V8.0 (BD Biosciences) was used to quantify the distribution of cells in each cell cycle phase: sub-G1 (dead cells), G1, S and G2/M. Results were displayed as the average of three separate experiments.

### Real-time proliferation analysis

Cell proliferation was monitored by the xCELLigence RTCA DP Analyzer (Roche) for at least 48 h after treatment, following manufacturer’s indications. This apparatus makes it possible to follow the cellular response to treatment in real-time using electrical impedance as the readout. The continuous monitoring of cell viability by the xCELLigence system allows us to distinguish between cell death and reduced proliferation [29]. Cells (5 × 10^3^ cells/well) were seeded into E-plates 16 (Acea Biosciences Inc.) and impedance was continuously recorded in 15 min intervals until the end of the experiment. Cell index (CI) values, derived from the measured impedances, were acquired by the RTCA Software V1.2 (Roche) and exported to Microsoft Excel for normalization of data of each single well to the first measurement after starting the treatment. Statistical analysis and graphical representation of data were performed by the Prism GraphPad Software V5.0 (GraphPad Software Inc., La Jolla, CA, USA). Data displayed in the graphs is the average value of three biological replicates, each consisting of two technical replicates.

### Cell number quantification, proliferation assay and detection of apoptosis

To assess proliferation after treatment by measuring the amount of newly synthetized DNA, cells were plated in a 96-well plate (5 × 10^3^ cells/well) and the Click-iT® EdU cell proliferation assay (Life Technologies) was used following the manufacturer’s instructions. Cells were incubated with 10 μM of the nucleoside analog EdU for 2 h and immediately fixed in 4 % formaldehyde and permeabilized. To detect apoptosis, cells were stained for 1 h at room temperature with anti-active-caspase-3 antibody, 1:600 (ab13847; Abcam) followed by goat anti-rabbit secondary antibody staining, Alexa Fluor® 488, 1:1000 (#A-11070; Life Technologies), for 1 h at room temperature. The total DNA was stained with Hoechst 33342 (Life Technologies) and used for quantifying the absolute number of cells present in the plate. Quantification of fluorescent cells that incorporated Hoechst 33342, EdU or were stained for caspase-3 was carried out by using the Operetta® High Content Imaging System equipped with the Harmony software (PerkinElmer Inc.). Fractions of EdU labeled cells were calculated based on Hoechst signal. Three independent experiments, with two internal replicates, were performed for each condition.

### Statistical analysis

All statistical analysis were performed by Prism GraphPad Software V5.0 (GraphPad Software Inc.) except for the association of copy number amplifications, detected by aCGH, with tumor thickness, which was calculated by the Mann–Whitney test in the R software environment for statistical computing. Detailed methods for the identification of CNAs from the aCGH data are provided in ref. [15]. The Mann–Whitney test was used to evaluate the association between MM thickness and copy number levels of TrkA derived from aCGH and genomic qPCR analysis. Pearson’s correlation coefficient was use to assess correlation between the aCGH copy number log2 ratio and the log2 of the qPCR fold changes of TrkA. Spearman’s correlation test was used to evaluate the correlation between TrkA copy number and mRNA expression data extracted from publically available resources: Cancer Cell Line Encyclopedia (CCLE, http://www.broadinstitute.org/ccle/home) and The Cancer Genome Atlas data (TCGA, http://www.cbioportal.org/index.do; ref. [30, 31]).

The Kaplan-Meier method and log-rank test were used to assess the difference in overall survival and metastatic outcome between TrkA-amplified patients and TrkA-diploid patients. One-way ANOVA test, followed by Tukey’s post-test to compare two groups, was performed to explore differences of proliferation rates in the xCELLigence proliferation assay. Student’s *t* test (two-tailed, unpaired) was used to compare means for all other statistical analyses. Results for cellular experiments are given as the mean of three independent experiments; p values were considered significant when lower than 0.05.

## Results

### Identification of TrkA amplification in MM patients

**Fig. 1:**
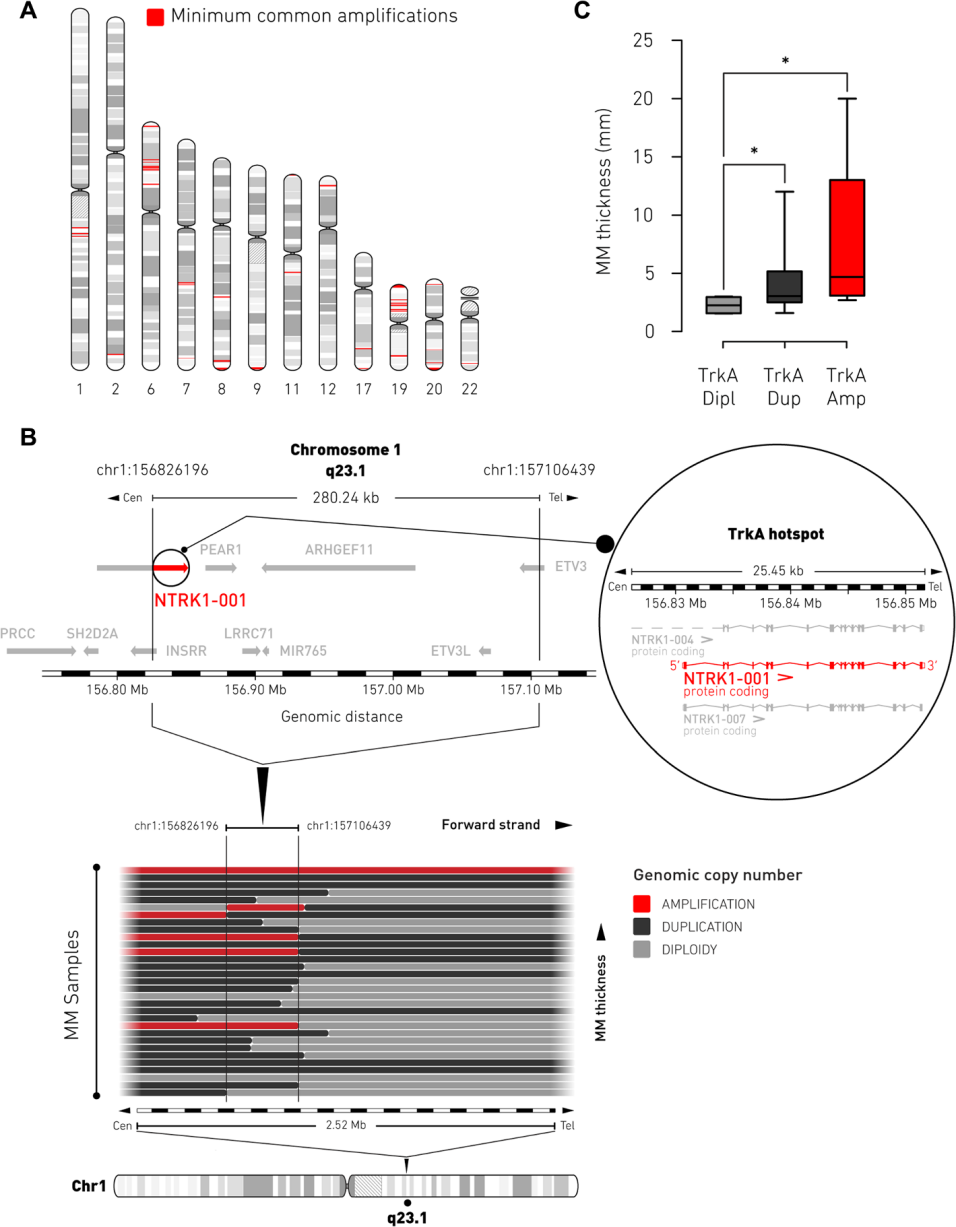
Identification of TrkA-1q23.1 genomic amplification in MM patients. **a**, hotspots of 40 minimum common amplifications (red) in primary MM genome, detected by aCGH across 31 patient samples, are plotted along their corresponding chromosome position and proportionally to the respective amplicon size. Detailed genomic information of hotspots is provided in Additional file 1: Table S2. **b**, schematic segmental gain profile within the 1q23.1 region (spanning ~2.5 Mb), as defined by aCGH, is represented with horizontal bars, each denoting the copy number status of an individual MM patient. MM samples with increasing primary tumor thickness are at the top (for details see Additional file 1: Table S4). Genomic amplifications are depicted in red. The black boundaries delineate the extent of the minimal common amplification (genomic coordinates chr1:156826196 to chr1:157106439). The graphical layout of the genes localized in the minimal amplification is based on the Ensembl release 75.37 of the human genetic map. The region of minimal common amplification extends over ~280 kb and retains the functional transcript of the *NTRK1* gene [GenBank: Y09028], NTRK1-001 (red and inset), which codes for TrkA protein. For each panel, the corresponding scale of genomic positions (in Mb) is indicated. **c**, box and whiskers graph showing the association of TrkA-1q23.1 minimal amplification and tumor thickness in primary MM samples analyzed by aCGH (*n* = 31; Mann-Whytney *U* test: *, *p* < 0.05). Dipl, diploid copy number; Dup, duplication; Amp, amplification

Genomic amplification is a potential indicator of oncogene activation. To identify candidate oncogenes that participate in melanomagenesis, we retrospectively analyzed 31 primary MM samples, previously characterized for genomic profiles with aCGH (GSE45354; ref. [15]), by exploring the association between genomic amplification and tumor thickness, a first-line clinical parameter of MM progression. Altogether, we detected 40 minimal common amplification hotspots over 12 chromosomes, consisting of average 5.7 amplicons per MM genome with a mean size of 0.47 Mb. A total of 994 unique genes are present within the amplicons, preferentially localized in 1q21–23, 6p21–25, 8q24, 19p13, and 20q13 (Fig. 1a and Additional file 1: Table S2). This produces a pattern similar to those observed in previous studies [18, 20], and supports the validity of our analysis.

Among the most frequently amplified loci identified in the MM genome, the 1q23.1 hotspot (amplified in 16 % of patients) had the strongest statistical association (Mann–Whitney *U* test: *p =* 0.03) with primary tumor thickness (Additional file 1: Table S2). This minimum common region of amplification displayed a characteristic profile of segmental gain, as defined by aCGH, that spanned over 280 kb (Fig. 1b), supporting the hypothesis of tumorigenic selective pressure. Analysis of correlation showed that tumor thickness proportionally increased in those primary tumors undergoing allele duplication (Mann–Whitney *U* test: *p =* 0.03) or amplification (Mann–Whitney *U* test: *p =* 0.03) of the 1q23.1 hotspot, compared to diploid samples (Fig. 1c). Median thickness of MMs that harbored the 1q23.1 amplification was 4.7 mm (range 1.6–20.0 mm), compared to 3.0 mm (range 2.5–12.0 mm) when the 1q23.1 locus is duplicated, and 2.3 mm (range 1.5–3.0 mm) of those MMs that maintain diploid 1q23.1. Therefore, we closely examined the genes localized in the 1q23.1 amplicon for a potential role in MM oncogenesis. Of the seven protein-coding genes and one miRNA gene present in the minimal common region of the same amplicon, the TrkA gene was the most promising candidate for driving segmental amplification within the 1q23.1 region in MM, based on its important role in melanocyte biology and known involvement in cancer. Interestingly, the minimal segmental alteration included only part of the long non-functional isoform (NTRK1-004) of the *NTRK1* gene [GenBank: Y09028] while it fully encompassed the entire functional isoform (NTRK1-001), which starts from a secondary transcription site and encodes for the canonical receptor tyrosine kinase TrkA (Fig. 1b). This observation may suggest the presence of a 5′ breakpoint occurring inside the *NTRK1* gene and localizing immediately upstream to the transcription start site of the functional isoform of TrkA.

### TrkA amplification associates with MM progression and negative patient outcome

**Fig. 2:**
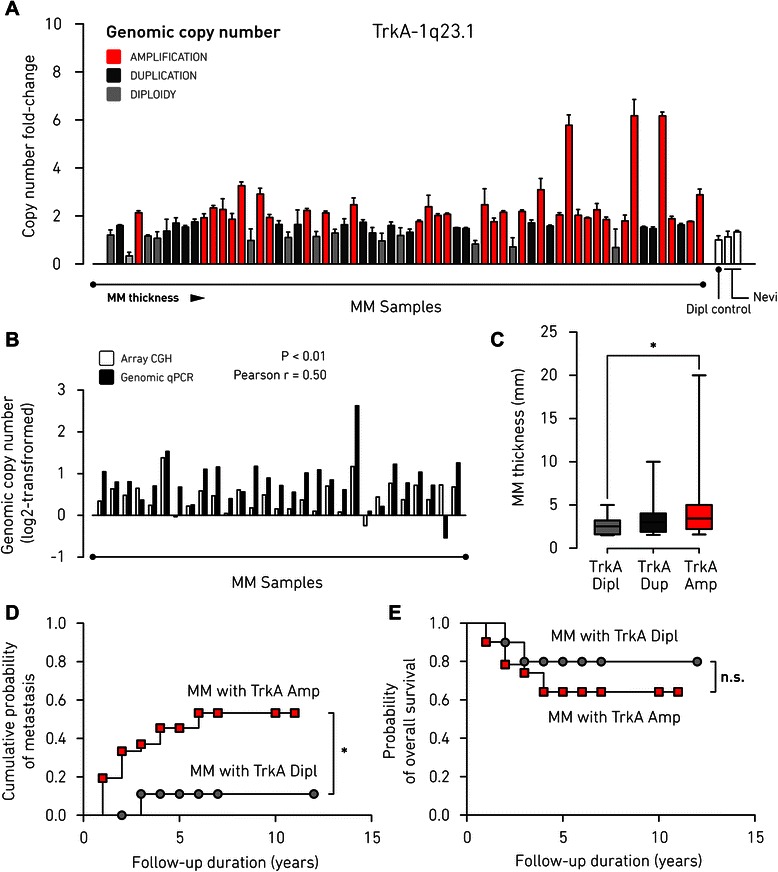
TrkA amplification associates with primary MM thickness and metastatic outcome. **a** genomic qPCR detection of copy number levels of TrkA gene in primary MM samples (*n* = 64), reported as fold-change over a diploid control of pooled healthy DNA (mean ± SD of *n* = 2 independent experiments, each of three replicates). Two additional samples of benign nevi were used as further accuracy control for diploidy. Samples are arranged according to increasing tumor thickness. Genomic amplification is depicted in red. **b** comparison of TrkA copy number levels for 29 primary MM samples from the aCGH dataset showing significant correlation between aCGH and qPCR. Log2-transformed fold changes (sample/control) of qPCR results are plotted with the corresponding aCGH log2 ratio mean values (Pearson’s correlation: *p* < 0.01; Pearson’s correlation coefficient, *r* = 0.5). **c** box and whiskers graph of the association between TrkA amplification and tumor thickness in primary MM samples analyzed by genomic qPCR (*n* = 64; Mann-Whytney *U* test: *, *p* < 0.05). **d** Kaplan–Meier curves for metastasis free survival in patient cohorts with TrkA amplification (*n* = 32) or TrkA diploidy (*n* = 12), as detected by genomic qPCR of primary MM genome (*, *p* < 0.05 by log-rank test). **e** Kaplan–Meier curves of overall survival for patients with TrkA amplification (*n* = 32) or diploid TrkA (*n* = 12), as detected by qPCR on primary MM genome (n.s., not statistically significant by log-rank test). Dipl, diploid copy number; Amp, amplification

To validate the discovery of the TrkA-1q23.1 amplicon as a potential hotspot associated with tumor progression, we performed genomic qPCR in a cohort of 64 primary MMs, including 29 samples previously analyzed by aCGH (we were able to perform qPCR only on 29 samples of the 31 included in the aCGH set, because of the limited amount of starting gDNA). This analysis revealed that TrkA amplification is a frequent event (50 % of the patients) in MM (Fig. 2a). The accuracy of our analysis was tested by comparing the aCGH data (Additional file 1: Table S3) to the results obtained by genomic qPCR (Additional file 1: Table S4): for each sample, the qPCR copy number fold changes (sample/diploid control) were converted to log2 values for direct comparison with the mean values of log2 ratios from aCGH signals. The directions of copy number changes were consistent for 27 samples out of 29, showing good concordance between the two methods (Fig. 2b). Besides, as a control for experimental reliability, we performed the same analysis on the *CDKN2A* gene, which is a major marker of MM-associated CNAs [9], obtaining results in agreement with what expected from the literature (Additional file 2: Figure S1).

Next, we examined the association of the TrkA copy number measured by genomic qPCR with the MM thickness and found that primary tumors with TrkA amplification were significantly thicker (*p =* 0.02) compared to tumors with diploid TrkA (Fig. 2c). Samples were then verified for the association of clinical outcomes with copy number status (with or without amplification) of TrkA, by using Kaplan–Meier analysis. Patients presenting TrkA amplification showed earlier recurrence of metastasis to distant organs than those with diploid TrkA, as detected by qPCR (Fig. 2d; hazard ratio = 0.30; 95 % confidence interval = 0.09–0.98; log-rank test, *p =* 0.046). Patients with TrkA amplification also showed a tendency to survive less relative to TrkA-diploid patients (Fig. 2e), although the difference in overall survival was statistically not significant (hazard ratio = 0.54; 95 % confidence interval = 0.14–2.07; log-rank test, *p =* 0.37). Taken together, these results confirm our findings in the discovery set of array CGH, giving indication of TrkA amplification as a specific oncogenic event occurring in MM that correlates with the aggressiveness of the primary tumor.

We tried to substantiate our hypothesis through the analysis of public resources. By looking at The Cancer Genome Atlas (TCGA) data available through the cBioPortal (http://www.cbioportal.org/index.do; ref. [30, 31]), the TrkA gene is recurrently altered (14 % of 278 reported tumor samples with RNA-seq and CNA data) in MM, via amplification, mRNA level upregulation, and missense mutations (Additional file 2: Figure S2A). Cases with alterations tend to have the worse prognosis (median month survival of 35.91) compared to cases without TrkA alterations (median month survival of 65.87), although the difference is not statistically significant (Additional file 2: Figure S2B).

### Reconstitution of TrkA signaling blocks proliferation of MM cells *in vitro*

Although histological immunostaining of TrkA has been associated with the clinical outcome of MM [32], very little is known about its molecular function in MM cells. Thus, we first confirmed that genomic copy number gain of TrkA was also present in cell lines derived from aggressive tumors (Additional file 2: Figure S3A). However, when we checked the expression levels of TrkA we found that, on the contrary to what expected, endogenous protein and mRNA levels were basically undetectable in these MM cell lines, while endogenous TrkA expression was clearly distinguishable in the positive control (PC12 pheochromocytoma rat cells) and, to a certain extent, also in normal human melanocytes (Additional file 2: Figure S3B and S3C). To confirm this finding we surveyed the data available at the Broad-Novartis Cancer Cell Line Encyclopedia (CCLE, http://www.broadinstitute.org/ccle/home) and found that the log2 mRNA levels of TrkA are indeed quite low (median log2 = 3.8; CI: 3.8–4.0), although a fraction of these cells lines show gain or amplification of the TrkA locus (Additional file 1: Table S5). This observation brought to the hypothesis that the contribution of TrkA overexpression (acquired through genomic gain) to the initial progression of the primary tumor might be negatively selected afterwards (by down-regulating gene expression), as it is reflected in our cell lines derived from advanced MMs. Analysis of CCLE cell line data revealed that TrkA mRNA levels and genomic amplification are indeed not correlating (Additional file 2: Figure S2C; Spearman r = 0.080). As well, we were not able to detect any significant correlation between TrkA mRNA and copy number levels in tumor samples collected by TCGA at the cBioPortal (Additional file 2: Figure S2D; Spearman r = 0.086) and listed in the Additional file 1: Table S6.

**Fig. 3:**
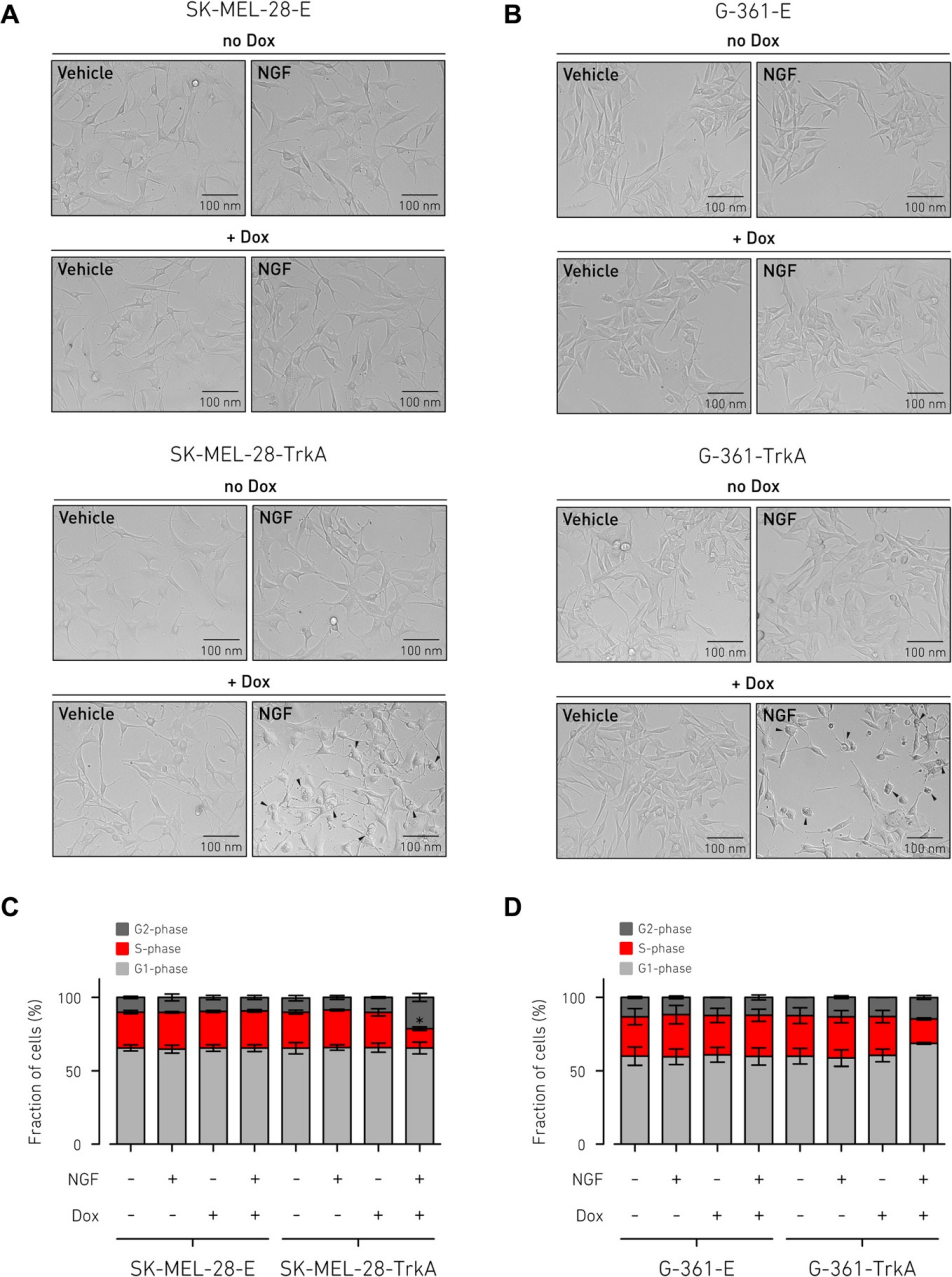
Activation of NGF-TrkA signaling induces cell cycle arrest of MM cells. **a** and **b**, representative images showing the morphology of MM cells upon TrkA ectopic expression and following activation by NGF. Stably transduced cells with either doxycycline-inducible vector (SK-MEL-28-TrkA and G-361-TrkA) or empty vector (SK-MEL-28-E and G-361-E) were incubated for 48 h with or without doxycycline (500 ng/ml) and next treated with NGF (100 ng/ml) or vehicle for 24 h in 2 % FBS medium. Images were obtained using phase-contrast microscopy from four independent experiments. Arrowheads indicate cells displaying distinctive morphological changes of oncogene-induced type growth arrest of intracellular vacuolization and cell shrinkage. **c** and **d**, cell proliferation of SK-MEL-28-E/TrkA and G-361-E/TrkA was tested by cell-cycle assay performed using DNA staining with propidium iodide and FACS analysis. Cells were incubated with or without doxycycline (500 ng/ml) for 48 h and then stimulated with NGF (100 ng/ml) for 24 h or vehicle treated. Bar graphs represent frequency of cell-cycle phases as the mean ± SD of *n* = 3 independent replicates (Student’s *t* test: *, *p* < 0.05 compared to vehicle control). Dox, doxycycline

To further explore our hypothesis, we reconstituted NGF-TrkA signaling by controlled expression of TrkA under a doxycycline-dependent promoter and NGF administration in two of the MM cell lines previously tested: SK-MEL-28 and G-361 (Additional file 2: Figure S3D). Expression of TrkA was induced for 48 h, followed by 24 h of NGF stimulation. Interestingly, we observed that addition of NGF caused dramatic morphological changes of MM cells transduced with the TrkA-inducible system (SK-MEL-28-TrkA and G-361-TrkA) only upon activation of TrkA expression by doxycycline, in comparison with the same cells in the absence of doxycycline or the empty-vector controls (SK-MEL-28-E and G-361-E), as shown in Fig. 3a and b. This phenotype, exclusively dependent on the activation of the NGF-TrkA axis, became visible early after treatment, reaching its peak at 8 to 24 h, and consisted in a conspicuous intracellular vacuolization and cell shrinkage. Cell cycle analysis revealed that this phenomenon was accompanied by proliferation arrest, resulting from a reduction of the S-phase cell population of MM cells expressing TrkA upon NGF treatment, again relative to the empty vector transduced cells or doxycycline untreated cells. Block of cell cycle was especially marked for the SK-MEL-28-TrkA cell line, experiencing an increase (*p =* 0.03) of the G2 fraction (Fig. 3c), while the G-361-TrkA cell line showed a moderate increase (*p =* 0.07) of the G1-phase fraction (Fig. 3d). All together, these observations are consistent with a phenotype of checkpoint-guided inhibition of cell proliferation as a consequence of oncogene-induced growth arrest.

### MAPK and AKT mediate opposite effects during proliferation arrest of MM cells induced by NGF-TrkA signaling

**Fig. 4:**
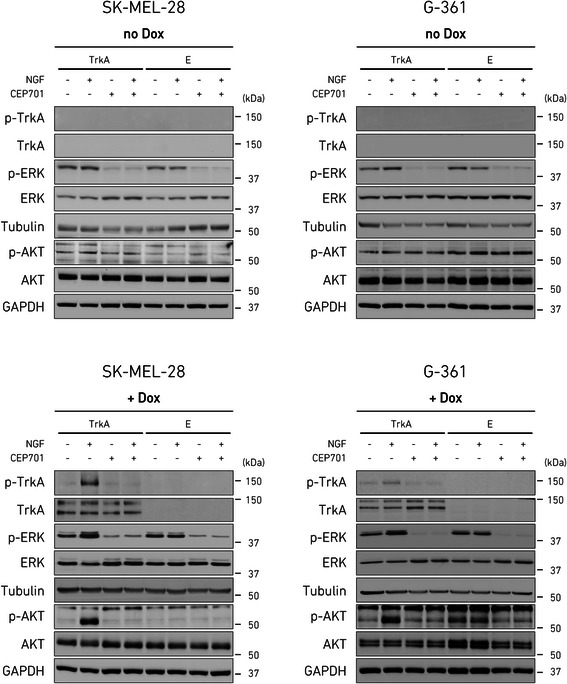
Activation of MAPK and AKT downstream to NGF-TrkA signaling in MM cells. SK-MEL-28 and G-361 cells, stably transduced with doxycycline-inducible TrkA-vector or empty vector were incubated for 48 h with vehicle (DMSO) or doxycycline (500 ng/ml) in FBS-free medium. Cell extracts were collected at 15 min post-stimulation with vehicle or NGF (100 ng/ml) and subjected to Western blotting using the indicated antibodies. Anti-β-tubulin was used as loading control for TrkA and ERK; anti-GAPDH was used as loading control for ATK. The protein markers in kDa are estimated from the molecular weight standard. Images are representative of *n* = 3 experiments. Dox, doxycycline; E, empty vector

**Fig. 5:**
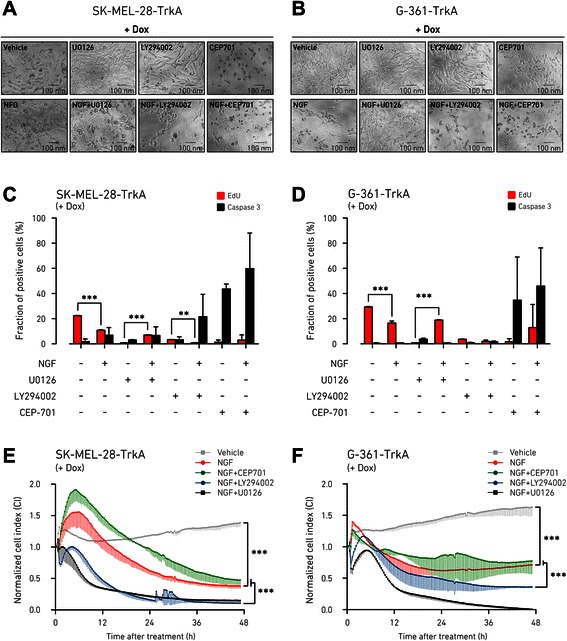
Proliferation arrest promoted by NGF-TrkA signaling in MM cells is enhanced after AKT pathway inhibition and dampened by the inhibition of MAPK pathway. **a** and **b**, stably transduced MM cells with doxycycline-inducible TrkA-vector (SK-MEL-28-TrkA and G-361-TrkA) were incubated for 48 h in 2 % FBS medium with doxycycline (500 ng/ml) and next treated with vehicle (DMSO), MAPK pathway inhibitor U0126 (5 μM), AKT pathway inhibitor LY294002 (25 μM), or the broad range receptor kinase inhibitor CEP-701 (10 μM), in the presence or absence of NGF (100 ng/ml) for 24 h in 2 % FBS medium. Images of morphological changes induced by treatment are representative of three independent experiments and were obtained using phase-contrast microscopy from cells growth in 96-well plate. **c** and **d**, cell proliferation or apoptosis of 48 h doxycycline-induced (500 ng/ml) SK-MEL-28-TrkA and G-361-TrkA cells, was measured 24 h post-treatment with NGF or indicated kinase inhibitors (as above), by using Click-iT EdU cell-proliferation assay or caspase-3 immunostaining, respectively. Images were acquired and quantified by the Operetta High Content Imaging System and cell count reported as fraction over total cell number (mean ± SD of *n* = 3 independent replicates; Student’s *t* test: **, *P* < 0.01; ***, *P* < 0.001). **e** and **f**, proliferation of SK-MEL-28-TrkA and G-361-TrkA cells, pre-induced with doxycycline (500 ng/ml) for 48 h in 2 % FBS medium, was monitored real-time by using the xCELLigence system. Cells were then maintained for 48 h in 2 % FBS medium with NGF (100 ng/ml) in the presence or absence of kinase inhibitors U0126 (5 μM), LY294002 (25 μM), CEP-701 (10 μM) and compared to vehicle control. Relative cell proliferation was measured by cell index (CI) and normalized at the beginning of treatment. Error bars are showed as SD below the trend-line of the mean from three independent biological replicates, each consisting of two internal replicates. One-way ANOVA was performed followed by Tukey’s post-test (***, *P* < 0.001). Dox, doxycycline

The MAPK and AKT pathways are two major effectors of NGF-induced TrkA signaling in different cell models [1], although this function has not been elucidated for MM cells yet. Our data showed that short stimulation (15 min) with NGF could induce phosphorylation of TrkA along with activation of ERK1/2 (p42/p44 MAPK) and AKT1 kinases over the basal levels in the MM cell lines SK-MEL-28-TrkA and G-361-TrkA, previously prompted by doxycycline to express TrkA (Fig. 4). This observation indicated that both ERK and AKT were downstream kinases to NGF-TrkA signaling in MM cells, although ERK showed a sustained basal state of endogenous phosphorylation, while in a dose–response assay (Additional file 2: Figure S4) AKT phosphorylation seemed to be more dependent on NGF-TrkA activation. Hence, we wanted to examine the effects of specific inhibition of MAPK and AKT signaling during a prolonged period of time and test if these two pathways may have a role in the proliferation arrest phenotype we observed in the presence of active NGF-TrkA signaling. Cells were incubated with doxycycline for 48 h to induce TrkA expression before the 24 h treatment with kinase inhibitors. As expected, selective inhibition of ERK or AKT basal activity by the respective upstream inhibitors U0126, which blocks mitogen-activated protein kinase-kinase (MEK), or LY294002, which blocks phosphatidylinositol 3-kinase (PI3K), induced a visible change in cell viability compared to vehicle control, although it did not affected substantially the total cell number (Fig. 5a and b; Additional file 2: Figure S5B). Inhibition of broad receptor-tyrosine-kinase activity by CEP-701 induced instead a massive cell death, possibly due to generalized block of cellular signaling; therefore we used treatment with CEP-701 as a general cell death control. Inhibition of the AKT pathway in the presence of NGF apparently promoted a further increase in cell loss compared to the LY294002 treatment alone (SK-MEL-28-TrkA, *p =* 0.002; G-361-TrkA, *p =* 0.03), suggesting that AKT was not responsible for the cell proliferation arrest observed after activation of NGF-TrkA signaling, but instead could mediate a pro-proliferative stimulus downstream to the NGF-TrkA pathway (Fig. 5a and b; Additional file 2: Figure S5A). On the contrary, inhibition of the MAPK pathway in response to NGF-TrkA activation had no significant incremental effects over U0126 alone, but rather seemed to partially rescue cell viability, as compared to NGF stimulation (SK-MEL-28-TrkA, *p =* 0.01; G-361-TrkA, *p =* 0.0004), indicating that MAPK could be responsible for the anti-proliferative action of NGF-TrkA signaling (Fig. 5a and b; Additional file 2: Figure S5A). Treatment with NGF produced no effects when TrkA was not expressed, in the SK-MEL-28-E and G-361-E cells (Additional file 2: Figure S5). These phenotypic observations gave a first indication that MAPK and AKT might mediate different signaling downstream to the NGF-TrkA axis. To investigate if the effects due to inhibition of the NGF-TrkA downstream kinases were directly associated with cell proliferation or apoptosis, we performed EdU incorporation, which stains cells in active S-phase replication, and caspase-3 activity detection assay. First we observed that, independently from active NGF-TrkA signaling, inhibition of the MAPK or AKT pathway blocked basal proliferation of MM cells, without however inducing apoptosis (Additional file 2: Figure S6). Activation of NGF-TrkA signaling in SK-MEL-28-TrkA and G-361-TrkA cells led to a severe reduction in proliferation with marginal effects on caspase-3 activity only in SK-MEL-28-TrkA, confirming that the decrease in cell number we previously observed was due to lowered proliferation rate rather than increased apoptosis (Fig. 5c and d). Simultaneous addition of U0126 with NGF resulted in a strongly sustained proliferation (observed in both cell lines), compared to cells growing in the presence of U0126 alone (SK-MEL-28-TrkA, *p =* 0.0006; G-361-TrkA, *p =* 0.0002), suggesting a partial counteraction of the growth arrest effect, induced by the activation of NGF-TrkA signaling, when the MAPK cascade is blocked (Fig. 5c and d). On the opposite, addition of LY294002 to SK-MEL-28-TrkA cells treated with NGF induced a further decrease of cell proliferation (SK-MEL-28-TrkA, *p =* 0.005; G-361-TrkA, *p =* 0.08), along with an up-regulation of the apoptotic signal over NGF-untreated cells (particularly marked in SK-MEL-28-TrkA cells; Fig. 5c), indicating that AKT function could be crucial for mediating the pro-survival branch of the NGF-TrkA pathway. Results for empty vector controls and doxycycline untreated cells confirmed that these effects were induced only in the presence of active NGF-TrkA signaling (Additional file 2: Figure S5). To further validate that TrkA regulates the NGF-induced MAPK and AKT pathways with differential outcomes on MM cell proliferation we used impedance-based monitoring of cell proliferation/viability in time-lapse, whose readout correlates very well with cell amount. Results confirmed that AKT and MAPK pathways had an NGF-TrkA independent basal activity in MM cells: blocking both pathways results in a pronounced slowdown of cell proliferation, as compared to controls (Additional file 2: Figure S7). When NGF was added to doxycycline-activated SK-MEL-28-TrkA and G-361-TrkA cells, therefore reconstituting NGF-TrkA signaling, it induced a rapid decrease in cell proliferation, confirming our previous observations (Fig. 5e and f). Concomitant inhibition of the AKT pathway in the presence of NGF determined an even more pronounced proliferative reduction in both cell lines, although to a greater extent in SK-MEL-28-TrkA cells (Fig. 5e), as compared to NGF + U0126 treated cells and NGF treatment alone (Fig. 5e and f). On the contrary, inhibition of the MAPK pathway slightly delayed the decrease of cell proliferation induced by NGF-TrkA signaling (Fig. 5e and f). Taken together, these results indicate that both the MAPK and the AKT pathways participate in maintaining basal proliferation of MM cells. When NGF-TrkA signaling is activated, MM cells enter a rapid proliferative arrest (without induction of apoptosis). However, the downstream AKT pathway is mainly required to sustain proliferation and survival (inhibiting AKT following NGF-TrkA stimulus accentuates cell lost and growth arrest), while the MAPK pathway may also have divergent functions and mediate an anti-proliferative signaling in response to NFG-TrkA activation (cell viability is improved when MAPK is inhibited following the NGF-TrkA stimulus).

### MAPK induces up-regulation of p21^cip^ in response to NGF-TrkA signaling in melanoma cells

**Fig. 6:**
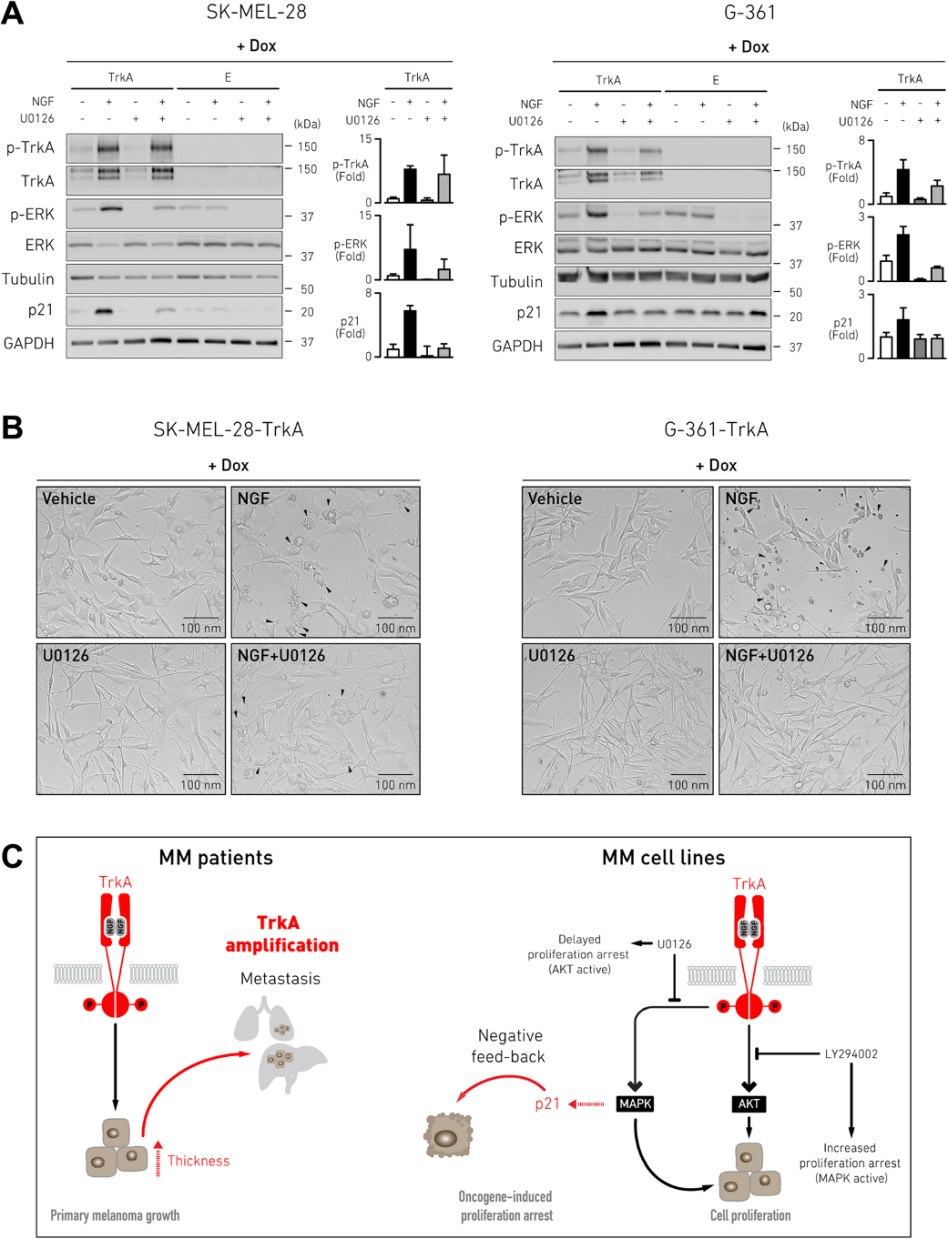
Induction of MAPK by NGF-TrkA signaling in MM cells results in increased expression of p21^cip1^. **a** MM cells (SK-MEL-28 and G-361), stably transduced with doxycycline-inducible TrkA-vector or empty vector, were incubated in 2 % FBS medium for 48 h with doxycycline (500 ng/ml) to induce ectopic TrkA expression and then treated for 24 h in 2 % FBS medium with vehicle, NGF (100 ng/ml), MAPK pathway inhibitor U0126 (5 μM), or NGF (100 ng/ml) coupled with U0126 (5 μM). Cell extracts were collected 24 h post-treatment ad subjected to Western blotting using the indicated antibodies. The protein markers in kDa are estimated from the molecular weight standard. Bar graphs show quantification of the intensity of the bands, compared to β-tubulin (phospho-ERK and phospho-TrkA) and GAPDH (p21^cip1^) loading control and to the relative untreated sample (set to 1). Data are expressed as mean ± SD of *n* = 3 experiments. **b** representative images obtained by phase-contrast microscopy from the same plate used for Western blotting, at 24 h after treatment. Arrowheads point at cells displaying morphological changes that are indicative of oncogene-induced growth arrest. **c** schematic model of our interpretation of the results: TrkA amplification in primary MM samples may be predictive of unfavorable patient outcome, correlating with increased primary tumor thickness and distant metastasis; in some circumstances, NGF-TrkA oncogenic signaling can couple with proliferation-arrest response that is mediated through p21^cip1^ up-regulation induced by MAPK, as suggested through selective inhibition of downstream MAPK or AKT pathways in MM cell line models. Dox, doxycycline; E, empty vector

It is known that prolonged activation of the MAPK pathway specifically mediates oncogene-induced growth arrest in several cancer models [11]. Although BRAF is the main mediator of this anti-proliferative response in MM [33], it is still not clear whether kinase membrane receptors can start these events. We investigated more in detail the molecular activity of the MAPK pathway downstream to NGF-TrkA signaling in doxycycline-induced SK-MEL-28 and G-361 cells, by using anti-phospho-TrkA and anti-phospho-ERK antibodies in Western blotting. First, we monitored the protein expression levels of known downstream targets of the TrkA-MAPK pathway that could be involved in cell cycle and proliferation of MM cells, including cyclin D1, p53, eIF4E, and p21^cip1^ (Additional file 2: Figure S8). Among this subset of genes, we were able to detect a consistent increase in p21^cip1^ protein levels in response to NGF stimulation of MM cells expressing TrkA, but not in unstimulated cells or empty vector controls. Since the anti-proliferative function of the MAPK pathway is primarily effected through upregulation of p21^cip1^ [33, 34], we blocked the MAPK cascade by incubating the cells with U0126 to observe consequent changes in p21^cip1^ expression. After 24 h, U0126 reduced to basal level the phosphorylation of ERK that was triggered by TrkA activation in the presence of NGF, while completely suppressing MAPK signaling in the absence of NGF (Fig. 6a). Protein levels of transduced TrkA were also increased upon NGF stimulation: this effect may be a consequence of a regulatory loop downstream to prolonged TrkA-kinase signaling, not observed instead after short stimulation (Additional file 2: Figure S4). The up-regulation of p21^cip1^ induced by the activation of MAPK via NGF-TrkA signaling was clearly prevented when U0126 was concomitantly added to the culture (Fig. 6a). As expected, we did not detect up-regulation of ERK phosphorylation and elevation of p21^cip1^ level downstream to NGF when cells were not induced by doxycycline to express TrkA (Additional file 2: Figure S9). Parallel analysis of cell morphology showed that, in the presence of active NGF-TrkA signaling, inhibition of the MAPK pathway by U0126 partially rescued the cell loss and vacuolization phenotypes that followed from NGF stimulation of doxycycline-induced SK-MEL-28-TrkA and G-361-TrkA cells (Fig. 6b). No changes were observed instead for doxycycline untreated cells and empty vector controls, confirming that these events were specifically dependent on NGF-TrkA signaling (Additional file 2: Figure S10). These results show a direct involvement of the MAPK pathway in response to sustained NGF-TrkA signaling to attenuate the tumorigenic phenotype of MM cells by promoting proliferation arrest and support a model of negative feedback loop that may act through the up-regulation of the p21^cip1^ tumor suppressor (Fig. 6c).

## Discussion

Although oncogenic activation of TrkA is traditionally associated with chromosomal translocation [6], gain and amplification of the genomic region 1q23.1, where the TrkA locus is located, may also occur at elevated frequency in a variety of cancers [20, 35]. Here, taking advantage of our MM panel of high-resolution copy number data [15], we could show, for the first time to our knowledge, that the amplification of TrkA may represent an important event contributing to primary MM oncogenesis. Across 40 amplicons detected in the genome of MM patients, TrkA-1q23.1 amplification was the most significantly associated with increased thickness of the primary tumor (Fig. 1). This finding indicates that TrkA signaling may be required for the onset of MM, when the tumor starts invading deeper in the skin. Precisely, the minimal amplification breakpoints created a hotspot that preserved the functional isoform of the gene encoding for the complete tyrosine-kinase receptor (Fig. 1b). As recently discovered, constitutive activation of TrkA is particularly common in spitzoid melanocytic lesions through coiled-coil fusion of the kinase domain [19]. The presence of multiple recombination sites spanning several TrkA exons [19] may reflect a biological property of the 1q23.1 region to be susceptible to genomic instability also in MM and undergo recurrent amplification as alternate mechanism of oncogenic selective pressure. This circumstance would match a pattern common to several known oncogenes, which can be often amplified, although preferentially activated by translocation [20, 36, 37]. Thus, the high incidence (50 % of MM biopsies) of TrkA amplification we reported in this study (Fig. 2a) might suggest that this is a prominent mechanism in primary MM to increase TrkA gene dosage during the initial phases of tumor formation. Next, we found that amplification of the TrkA locus specifically correlated with the metastatic course of MM patients (Fig. 2c). In agreement, benign nevi do not show histological expression of TrkA while phosphorylated TrkA levels increase significantly in primary MMs along with tumor thickness and the presence of phosphorylated TrkA in MM biopsies correlates with decreased overall survival [32]. In accordance with these previous histological data, the importance of NGF signaling in melanocyte biology [7], and its proved involvement in oncogenic pathways [4, 5], TrkA gene seems the most promising candidate for driving segmental amplification of the 1q23.1 region in MM, although we cannot exclude the possibility that the other genes (INSRR, PEAR1, LRRC71, MIR765, ARHGEF11, ETV3L, ETV3) within the 1q23.1 minimal common amplification could also participate in melanomagenesis. However, we did not find any relevant associations between the expression of these genes and MM clinical attributes when querying the public resource cBioPortal (data not reported).

Although it has been clearly shown that p75NTR, the cognate receptor of TrkA, is important for regulating the tumorigenic properties of MM cell lines [38, 39], direct evidences of TrkA involvement in MM cell behavior and melanomagenesis are lacking. Our study revealed that the induction of TrkA expression in MM cells following NGF stimulation significantly affects proliferation by blocking the cells in S-phase and provoking catastrophic vacuolization (Fig. 3), a phenomenon that is characteristic of oncogene-induced growth arrest [40]. However, NGF treatment of TrkA-expressing cells, while provoking rapid proliferation arrest, was not sufficient to promote complete cell death (Fig. 5), suggesting this anti-proliferative effect is only partial. As the NGF-TrkA signaling is classically implicated in MAPK and AKT activation [1] and TrkA overexpression stimulates both cascades in other tumor cells [41], we first verified that the two pathways are also activated downstream to NGF-TrkA signaling in MM cells (Additional file 2: Figure S4). Then we tested the effects of the inhibition of these two pathways on MM cell viability and proliferation. While both the MAPK and AKT pathways seemed to be important for sustained basal proliferation rate, inhibition of AKT activation specifically produced an additive effect to NGF-TrkA stimulation by enhancing cell loss (Fig. 5). On the contrary, treatment with an inhibitor of the MAPK pathway resulted in a partial recovery from cell proliferation arrest induced by NGF-TrkA activation (Fig. 5). These observations are in line with previous reports, where both MAPK and AKT are required for maintaining proliferation but specific suppression of AKT signaling completely abolishes the tumorigenic capacity of MM cells [42], while MAPK possesses a dual role of promoting proliferation on one side and enhancing anti-proliferative feedback on the other side [11, 33, 43].

It is generally accepted that oncogene-induced proliferation arrest may act through various mechanisms that culminate in MAPK overactivation [11, 44]. Here, we demonstrated that NGF is essential for prompting MAPK activity via TrkA phosphorylation in MM cells (Figs. 4 and 6). In these circumstances, activation of MAPK is associated with an increased expression of the p21^cip1^ protein, which is instead attenuated when MAPK signaling is inhibited (Fig. 6). Accordingly, addition of a MAPK inhibitor produced a visible abrogation of the vacuolization phenotype triggered by active NGF-TrkA signaling in MM cells (Fig. 6). These findings are similar to what previously shown in non-melanoma cells where apoptotic cell death is enhanced by MAPK activation after overexpression of TrkA [45–47]. Although abrogation of p16^ink4A^ function is the most common alteration that may lead to bypass the anti-proliferative control in MM [48], we showed here that in the absence of the p16^ink4A^ gene, as in our melanoma cell model, MAPK can induce a secondary mechanism of feedback protection that might go through p21^cip1^ up-regulation, which directly blocks MM cells at the S-phase checkpoint, as supported by previous evidence [33, 34]. Moreover, the MM cell lines investigated in our study were derived from advanced-stage MMs and harbor the BRAF^V600E^ constitutive-activating mutation, which could give predisposition to oncogene-induced growth arrest when MAPK is over-induced through alternative signaling [11, 49]. Importantly, proliferation arrest derived from constitutive stimulation of MAPK by BRAF is an intrinsic property of melanocytes and melanoma cells and is the pivotal mechanism to stop oncogenic progression in nevi, a type of benign melanocytic tumors [12, 50, 51]. Although, the overall expression of TrkA in MM biopsies correlates with worst prognosis, phosphorylated TrkA is detected in a considerably smaller fraction of metastatic MMs compared to the primary samples [32], suggesting that activation of TrkA signaling might be important for the early onset of the tumor and might undergo down-regulation once the tumor becomes metastatic. Further experiments would be required to substantiate this hypothesis. However, when we investigated public datasets we did not find any significant difference between TrkA mRNA expression in primary MMs versus metastatic MMs (data not shown). As well, expression of TrkA does not substantially differ in earlier compared to later MM stages (Additional file 2: Figure S2E).

The proposed dual function of TrkA in MM, acting in an oncogenic or in a tumor suppressor like mode, is similar to that of the melanocyte-specific protein MITF, which acts downstream to MAPK [51]. But, more significantly, a dual behavior of TrkA is particularly evident in neuroblastoma, a tumor of neural crest cell precursors [52]. In neuroblastoma patients, TrkA is initially gained and its overexpression is found mainly at lower stages and younger age at diagnosis [4]; in neuroblastoma cells instead, TrkA activation decreases proliferation via MAPK induction and induces anti-oncogenic response [46]. In the model depicted in Fig. 6c, we hypothesize that acquisition of TrkA amplification may arise early during melanomagenesis and contribute to the initial phases of oncogenic development, correlating with increased primary MM thickness. Primary tumors that present TrkA amplification show also greater association with distant organ metastasis. A multifaceted crosstalk between MAPK and AKT pathways downstream to NGF-TrkA signaling may then reflect different propensity of the MM cell to metastasize. In this context, oncogene-induced growth arrest *de facto* represents a barrier to tumor progression and consequently MM derived cell lines have bypassed this impediment. Reintroduction of active NGF-TrkA signaling may reimpose protective feedback mechanism by engaging p21^cip1^-dependent growth arrest via hyperactivation of MAPK.

## Conclusions

We identified TrkA as a candidate oncogene in MM that associates with clinical features of worst prognosis. We hypothesize that TrkA genomic amplification can promote tumor proliferation during the early phase of MM invasion, as indicated by the association with increased tumor thickness. Experiments in MM cells suggest that both the MAPK and AKT pathways may mediate this oncogenic propensity. At the same time, however, as reported for other tumor models, overactivation of the MAPK pathway in MM cells can induce a feedback protective effect against oncogenic growth, while the ATK pathway is mainly involved in stimulating proliferation downstream to NGF-TrkA signaling. Although the characterization of TrkA amplification in metastatic samples is missing and more detailed insights are required to understand the oncogenic potential of TrkA in normal melanocytes and MM cells with distinct molecular background, our observations identified a previously unknown association between TrkA function and MM development relying on TrkA amplification. Furthermore, our study emphasizes the dual role of MAPK signaling in promoting MM cell development on one side, and in inducing feedback proliferation arrest on the other side. These findings reinforce the current idea of exploiting physiological pathways promoting growth arrest for more effective cancer treatment [9, 13].

## Additional files

Additional file 1:
**Table S1** Histopathological and follow-up information of the 64 MM patients included in the study. **Table S2** Genomic amplification hotspots in MM and association test with tumor thickness, related to Figure 1. **Table S3** CGH array data of NTRK1 and CDKN2A for 31 primary MM specimens (from GEO Series GSE45354). **Table S4** Validation of the aCGH analysis by genomic qPCR, related to Figure 2 and Figure S1. **Table S5** Copy number and mRNA expression data for NTRK1 in CCLE melanoma cell lines, related to Figure S2. **Table S6** Copy number, mRNA expression, and clinical stage data for NTRK1 in TCGA melanoma samples, related to Figure S2.
Additional file 2: **Figure S1**Genomic copy number levels of CDKN2A in primary MM.**Figure S2**Bioinformatic analysis of TrkA mRNA expression and copy number in public database of MM cell lines and tumor samples.**Figure S3** Expression of TrkA in MM cell lines.**Figure S4**Dose-response activation of AKT and MAPK following stimulation of NGF-TrkA signaling in MM cell lines.**Figure S5**Morphological and quantitative analysis of MM cells in response to NGF-TrkA signaling.**Figure S6** Cell proliferation and apoptosis analysis of MM cells in the absence of active NGF-TrkA signaling following MAPK and AKT pathway inhibition.**Figure S7**Proliferation of MM cells in the absence of active NGF-TrkA signaling following MAPK and AKT pathway inhibition.**Figure S8** Analysis of MAPK downstream target expression following NGF-TrkA signaling in MM cells.**Figure S9** Analysis of MAPK activation and p21cip1 expression in MM cells in the absence of NGF-TrkA signaling following inhibition of MAPK pathway in MM cells.**Figure S10** Morphological analysis of MM cells in the absence of NGF-TrkA signaling activation and following inhibition of MAPK pathway.

## Abbreviations

NGF: Nerve growth factor
MM: Malignant melanoma
aCGH: Array comparative genomic hybridization
qPCR: Quantitative PCR
NTRK1: Neurotrophic tyrosine kinase receptor type 1
TrkA: TRK1-transforming tyrosine kinase protein
MAPK: Mitogen-activated protein kinase
PKB: Protein kinase B
p75NTR: p75 neurotrophin receptor
CNA: Copy number alteration
FFPE: Formalin-fixed and paraffin-embedded
gDNA: Genomic DNA
SG-PERT: SYBR Green I-based PCR-enhanced reverse transcriptase
