# Supplementary material for: TrkA is amplified in malignant melanoma patients and induces an anti-proliferative response in cell lines
Source: BMC Cancer. 2015 Oct 24;15:777. doi: 10.1186/s12885-015-1791-y (PMC4619539; doi:10.1186/s12885-015-1791-y)
Supplement: Additional file 2: — Figure S1Genomic copy number levels of CDKN2A in primary MM.Figure S2Bioinformatic analysis of TrkA mRNA expression and copy number in public database of MM cell lines and tumor samples.Figure S3 Expression of TrkA in MM cell lines.Figure S4Dose-response activation of AKT and MAPK following stimulation of NGF-TrkA signaling in MM cell lines.Figure S5Morphological and quantitative analysis of MM cells in response to NGF-TrkA signaling.Figure S6 Cell proliferation and apoptosis analysis of MM cells in the absence of active NGF-TrkA signaling following MAPK and AKT pathway inhibition.Figure S7Proliferation of MM cells in the absence of active NGF-TrkA signaling following MAPK and AKT pathway inhibition.Figure S8 Analysis of MAPK downstream target expression following NGF-TrkA signaling in MM cells.Figure S9 Analysis of MAPK activation and p21cip1 expression in MM cells in the absence of NGF-TrkA signaling following inhibition of MAPK pathway in MM cells.Figure S10 Morphological analysis of MM cells in the absence of NGF-TrkA signaling activation and following inhibition of MAPK pathway. [file 12885_2015_1791_MOESM2_ESM.pdf]

Figure S1

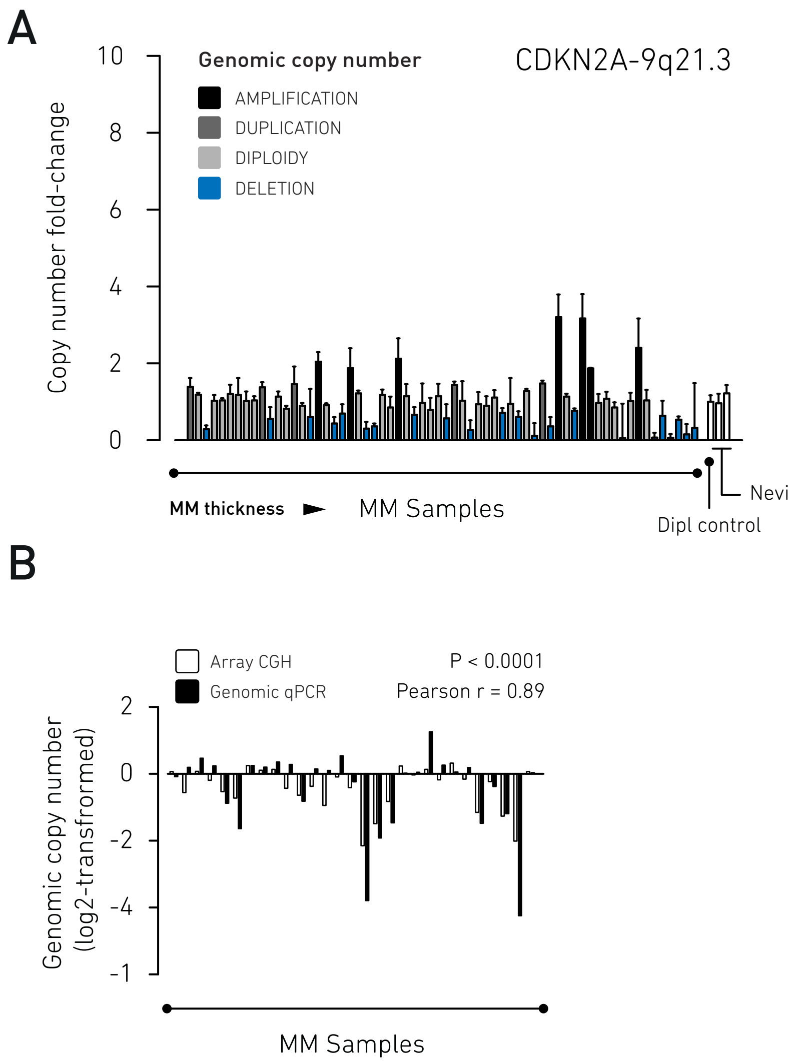
A
Copy number fold-change
10
8
6
4
2
0
Genomic copy number
AMPLIFICATION
DUPLICATION
DIPLOIDY
DELETION
CDKN2A-9q21.3
MM thickness
MM Samples
Nevi
Dipl control
B
Array CGH
Genomic qPCR
P < 0.0001
Pearson r = 0.89
Genomic copy number
(log2-transfromed)
2
0
-2
-4
-1
MM Samples

Figure S2

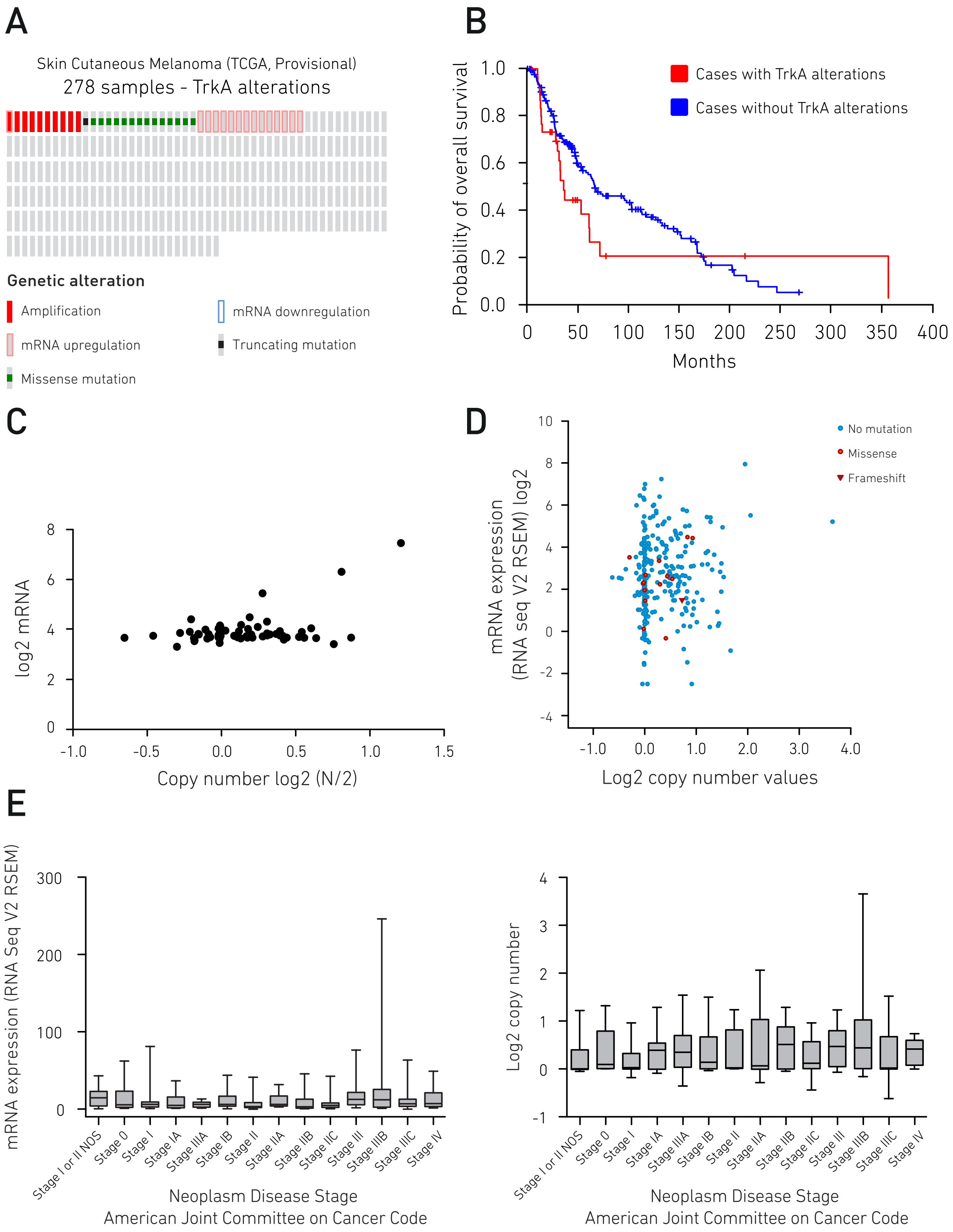
A
Skin Cutaneous Melanoma (TCGA, Provisional)
278 samples - TrkA alterations
Genetic alteration
Amplification
mRNA downregulation
mRNA upregulation
Truncating mutation
Missense mutation
B
Cases with TrkA alterations
Cases without TrkA alterations
Probability of overall survival
Months
C
log2 mRNA
Copy number log2 (N/2)
D
No mutation
Missense
Frameshift
mRNA expression (RNA seq V2 RSEM) log2
Log2 copy number values
E
mRNA expression (RNA Seq V2 RSEM)
Log2 copy number
Stage I or II NOS
Stage 0
Stage I
Stage IA
Stage IIIA
Stage IB
Stage II
Stage IIA
Stage IIB
Stage IIC
Stage III
Stage IIIB
Stage IIIC
Stage IV
Neoplasm Disease Stage
American Joint Committee on Cancer Code

Figure S3

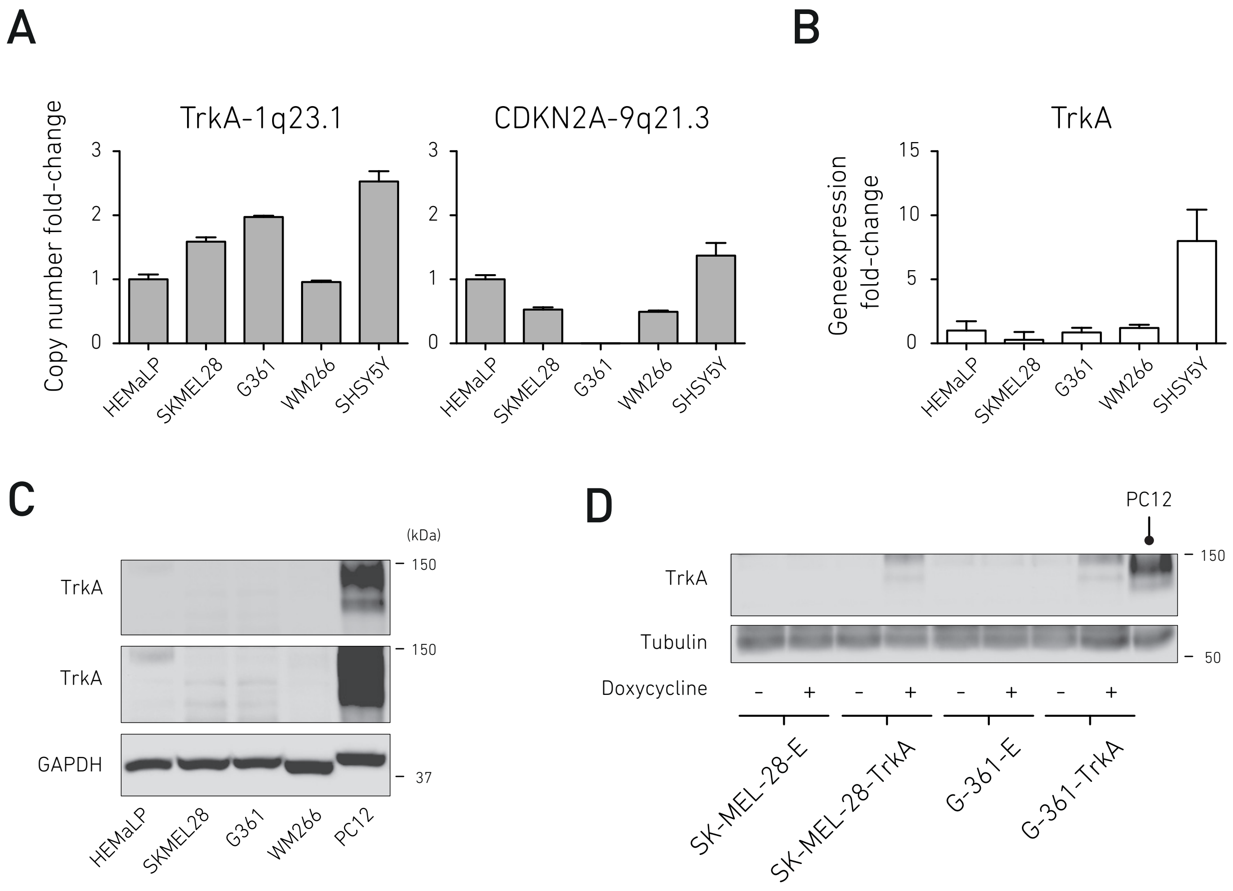
A
B
TrkA-1q23.1
CDKN2A-9q21.3
Copy number fold-change
HEMaLP
SKMEL28
G361
WM266
SHSY5Y
TrkA
Geneexpression fold-change
C
(kDa)
TrkA
TrkA
GAPDH
150
150
37
PC12
D
PC12
TrkA
Tubulin
Doxycycline
150
50
SK-MEL-28-E
SK-MEL-28-TrkA
G-361-E
G-361-TrkA

Figure S4

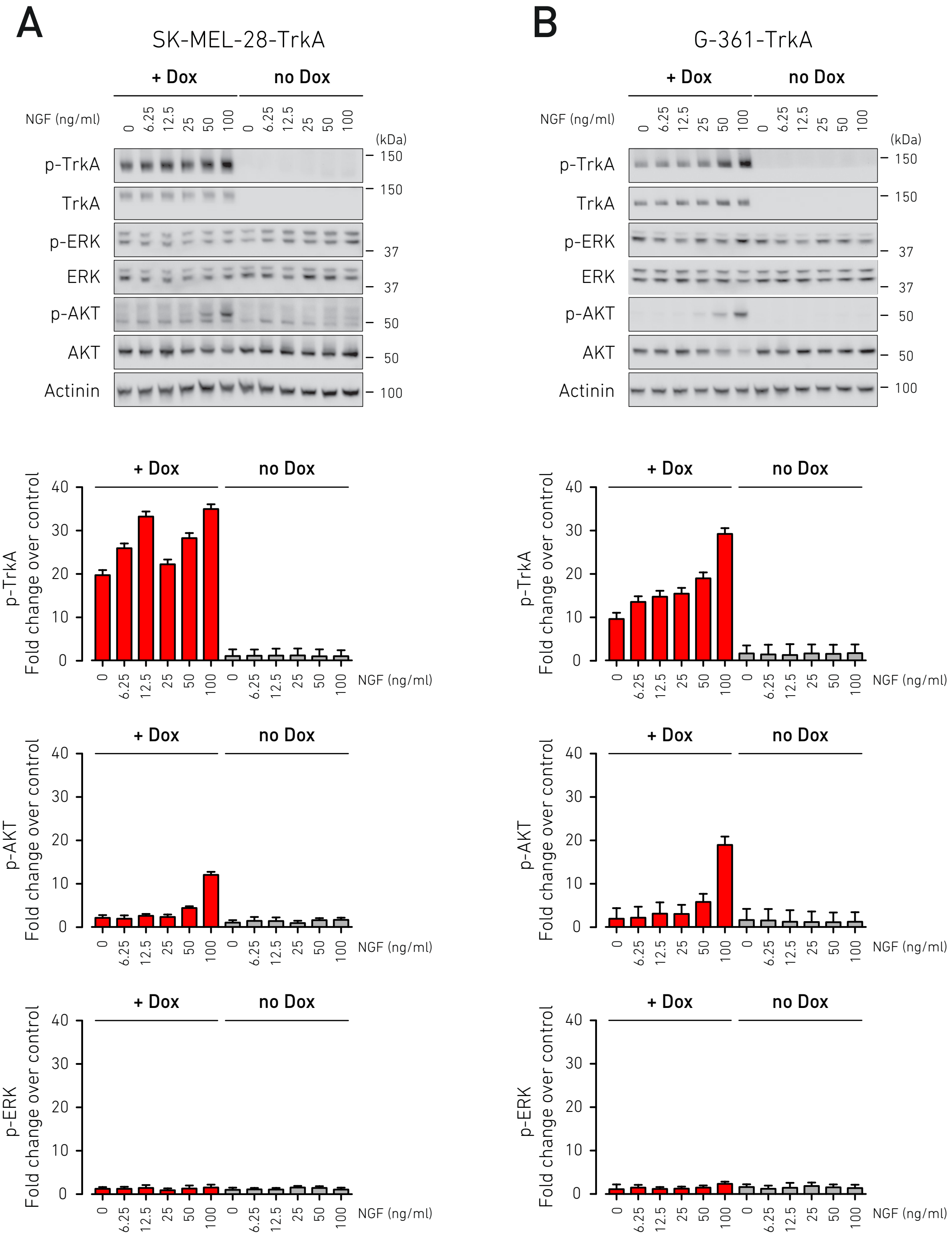

A
SK-MEL-28-TrkA
B
G-361-TrkA
+ Dox
no Dox
NGF (ng/ml)
0 6.25 12.5 25 50 100 0 6.25 12.5 25 50 100
(kDa)
p-TrkA
TrkA
p-ERK
ERK
p-AKT
AKT
Actinin
150
37
50
100
p-TrkA Fold change over control
p-AKT Fold change over control
p-ERK Fold change over control
0 10 20 30 40

Figure S5

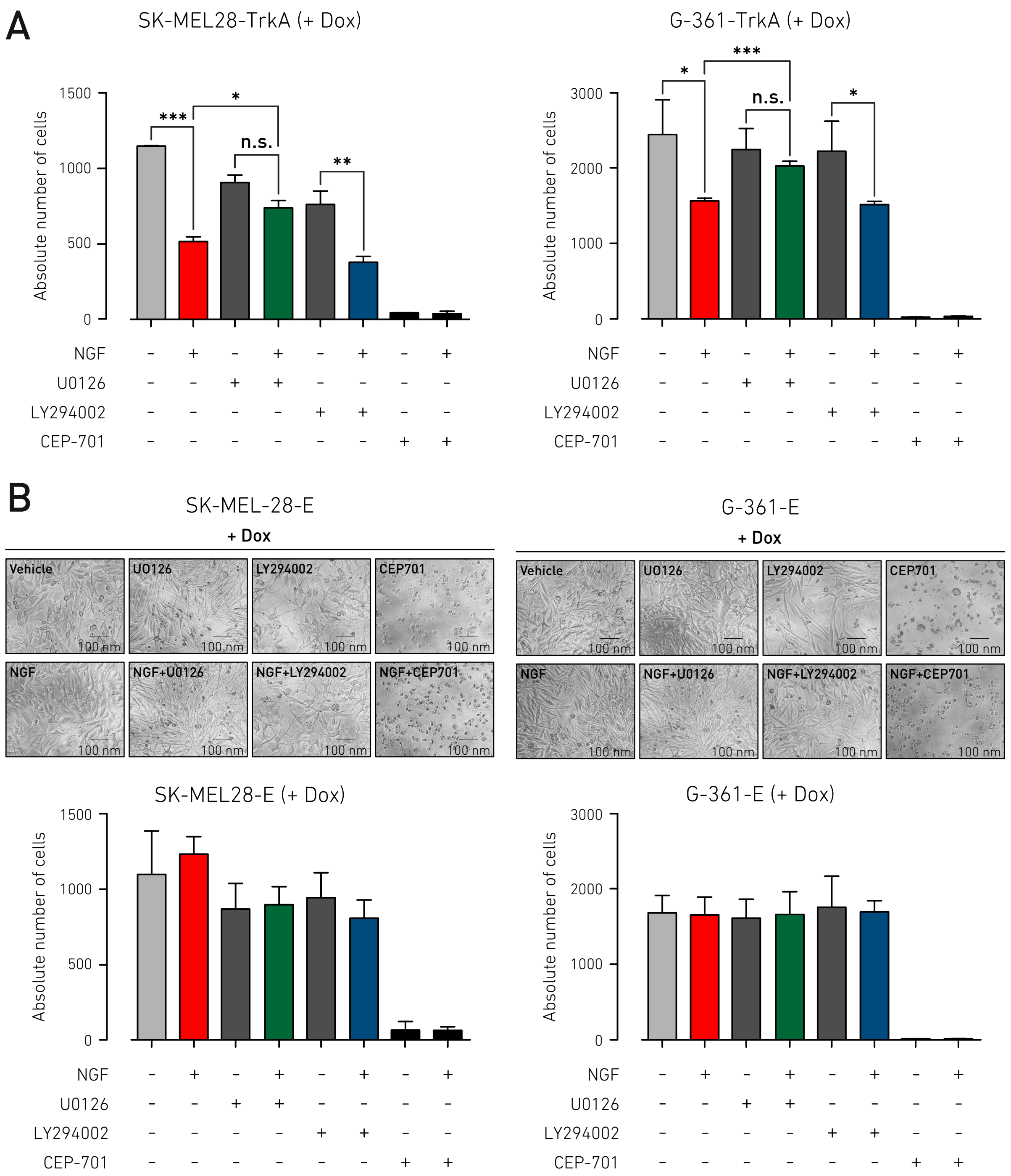

A
SK-MEL28-TrkA (+ Dox)
G-361-TrkA (+ Dox)
Absolute number of cells
***
*
n.s.
**
NGF
U0126
LY294002
CEP-701
B
SK-MEL-28-E
G-361-E
+ Dox
Vehicle
U0126
LY294002
CEP701
NGF
NGF+U0126
NGF+LY294002
NGF+CEP701
100 nm
SK-MEL28-E (+ Dox)
G-361-E (+ Dox)

Figure S6

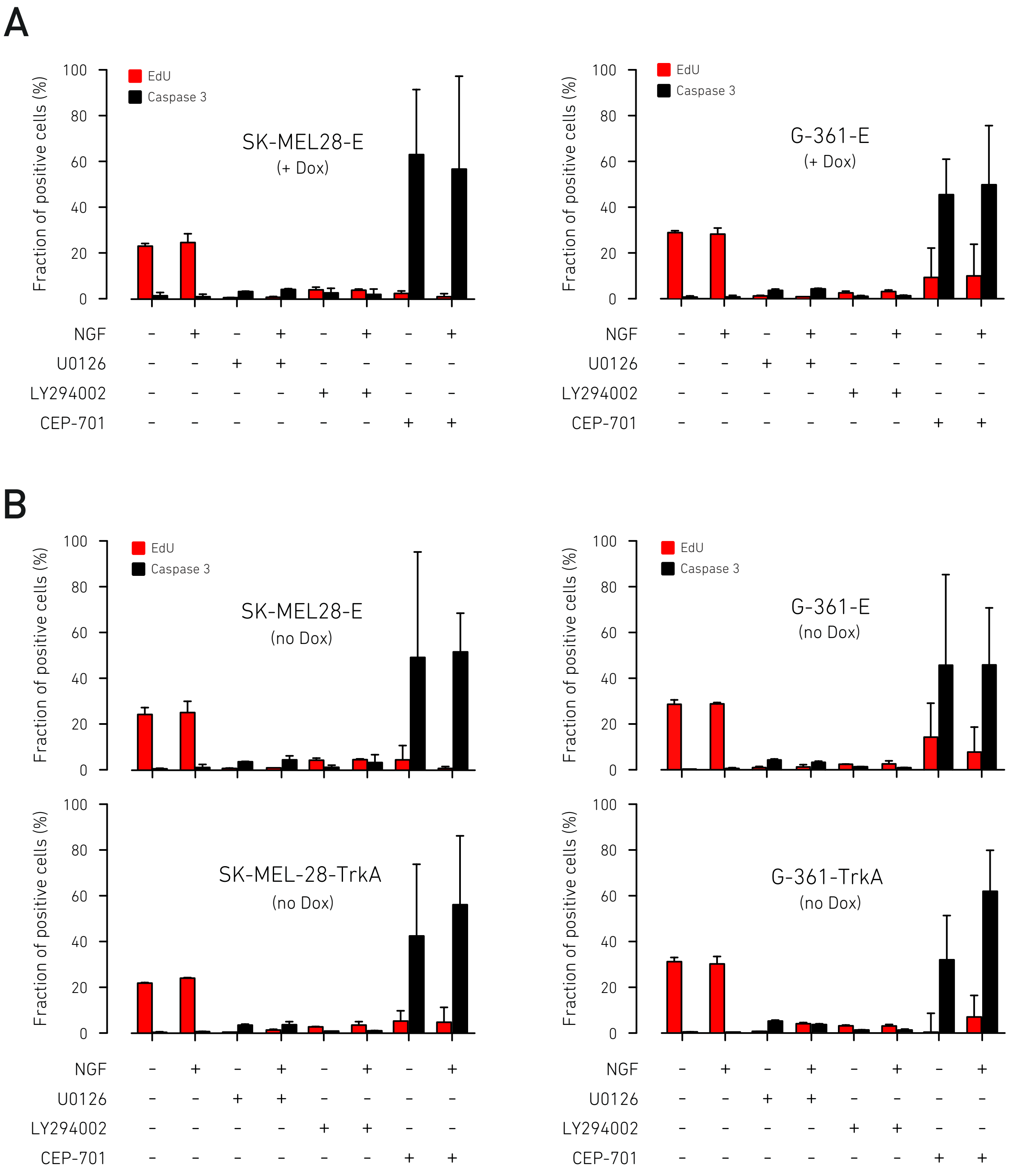
A
Fraction of positive cells (%)
100
80
60
40
20
0
EdU
Caspase 3
SK-MEL28-E
(+ Dox)
G-361-E
(+ Dox)
NGF - + - + - + - +
U0126 - - + + - - - -
LY294002 - - - - + + - -
CEP-701 - - - - - - + +
B
Fraction of positive cells (%)
100
80
60
40
20
0
EdU
Caspase 3
SK-MEL28-E
(no Dox)
G-361-E
(no Dox)
SK-MEL-28-TrkA
(no Dox)
G-361-TrkA
(no Dox)
NGF - + - + - + - +
U0126 - - + + - - - -
LY294002 - - - - + + - -
CEP-701 - - - - - - + +

Figure S7

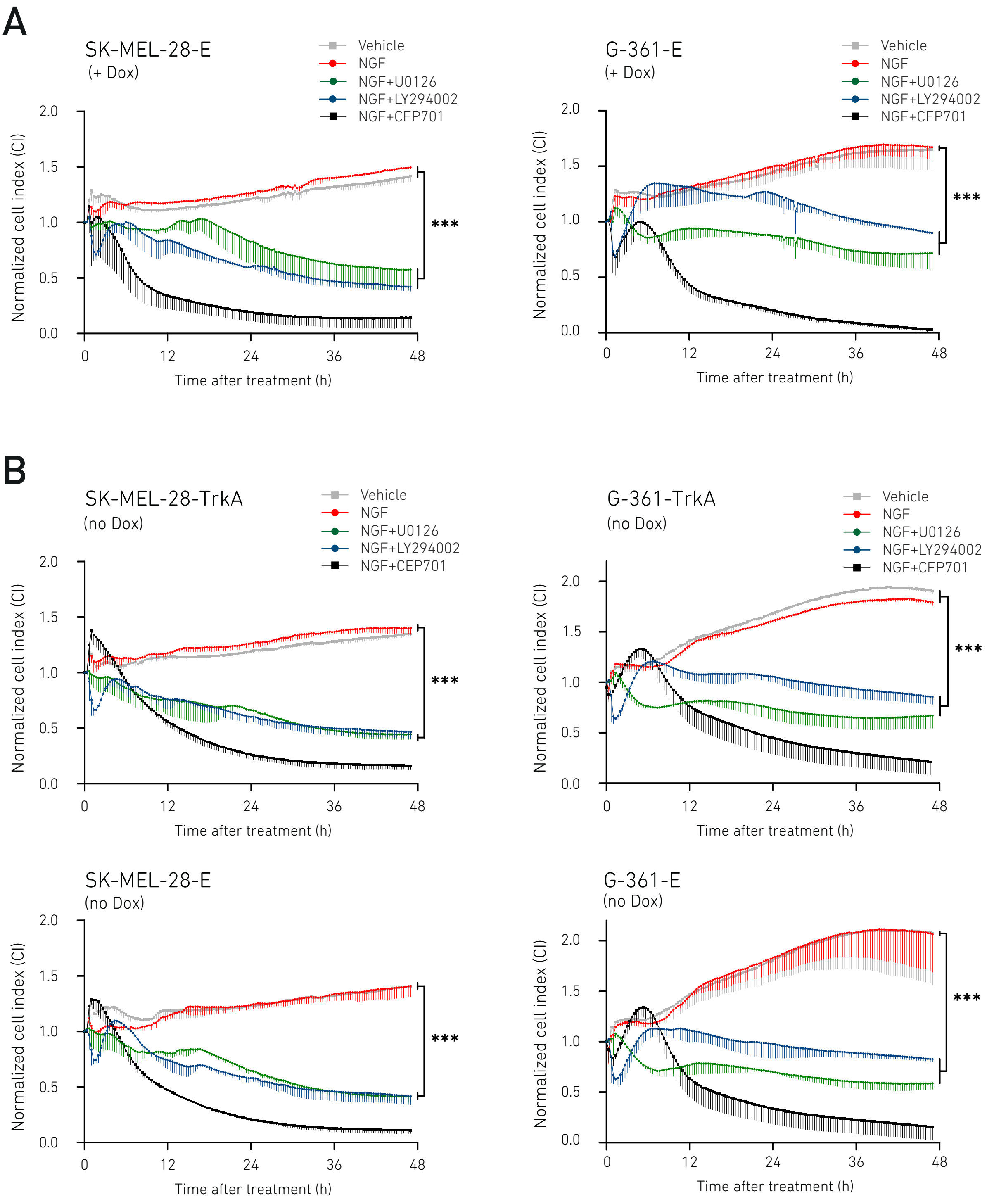
A
SK-MEL-28-E
(+ Dox)
G-361-E
(+ Dox)
B
SK-MEL-28-TrkA
(no Dox)
G-361-TrkA
(no Dox)
SK-MEL-28-E
(no Dox)
G-361-E
(no Dox)
Vehicle
NGF
NGF+U0126
NGF+LY294002
NGF+CEP701
Normalized cell index (CI)
Time after treatment (h)
0.0
0.5
1.0
1.5
2.0
0
12
24
36
48
***

Figure S8

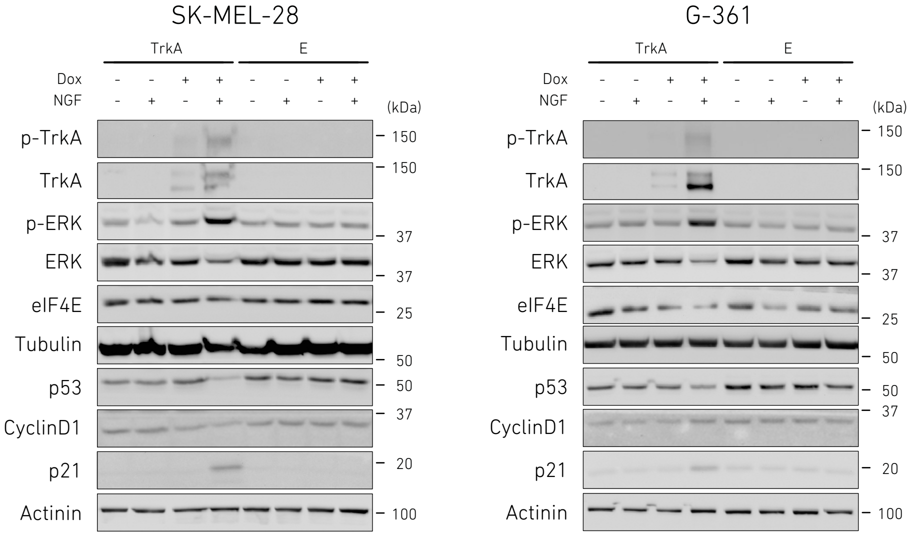
SK-MEL-28
G-361
TrkA
E
Dox
NGF
(kDa)
p-TrkA
TrkA
p-ERK
ERK
eIF4E
Tubulin
p53
CyclinD1
p21
Actinin
150
37
25
50
20
100

Figure S9

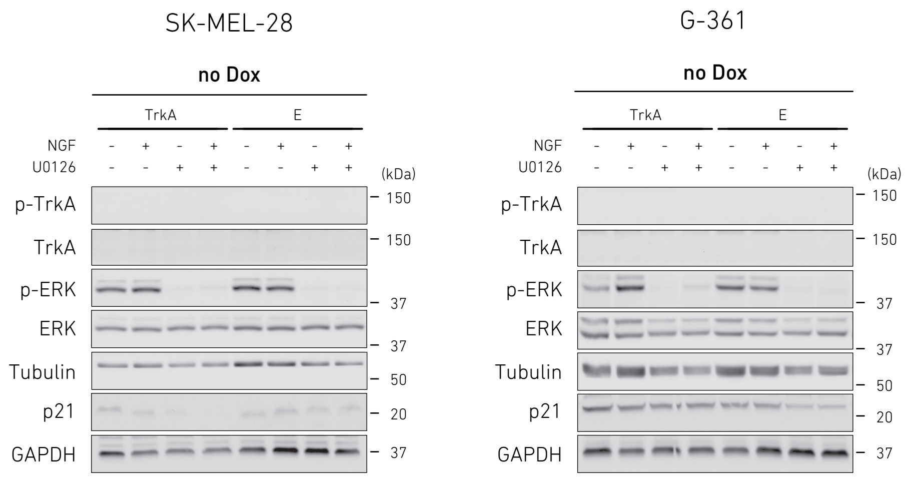

SK-MEL-28
G-361
no Dox
TrkA
E
NGF
U0126
(kDa)
p-TrkA
TrkA
p-ERK
ERK
Tubulin
p21
GAPDH
150
37
50
20

Figure S10

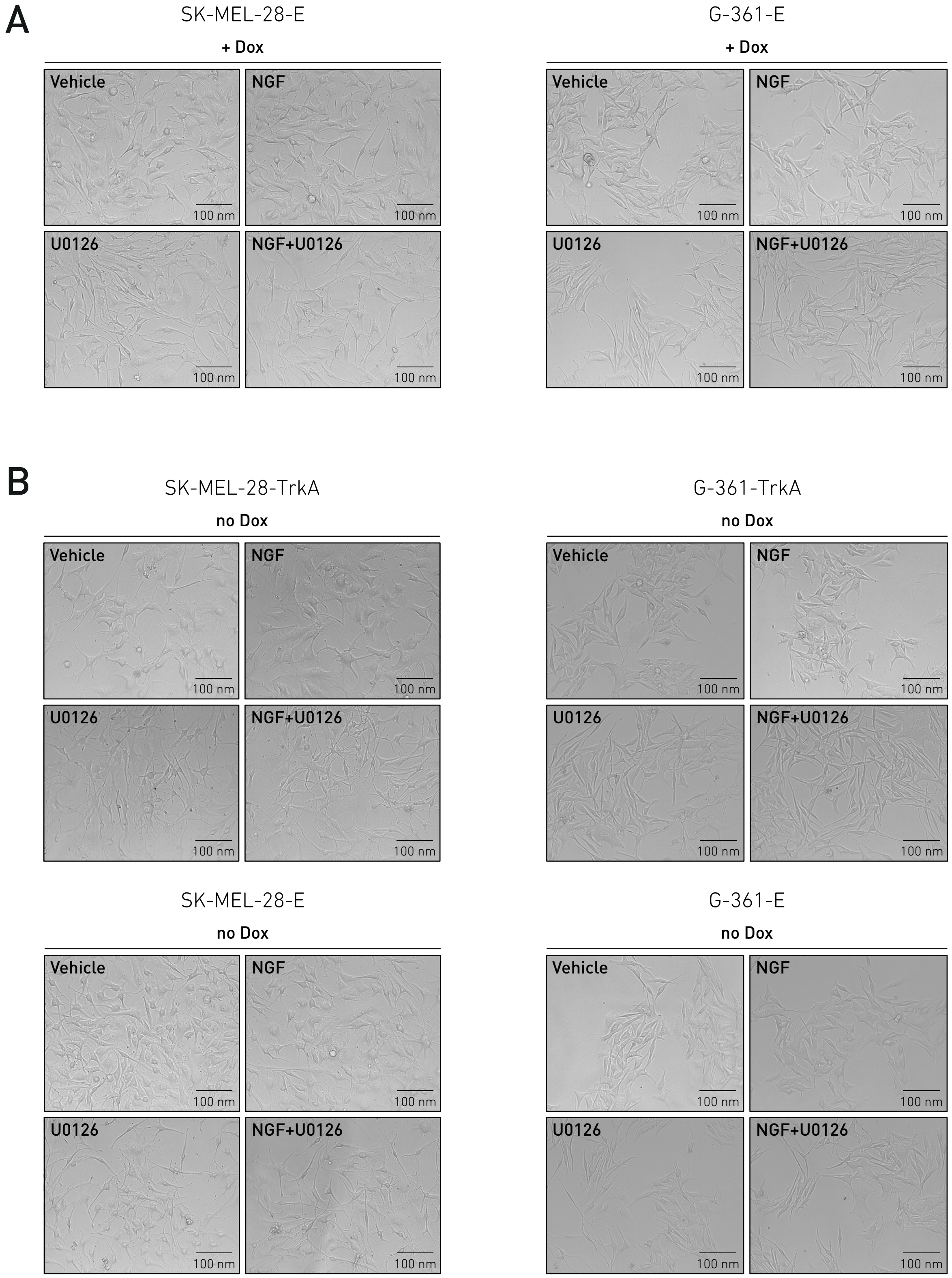
A
SK-MEL-28-E
+ Dox
Vehicle
NGF
U0126
NGF+U0126
G-361-E
+ Dox
Vehicle
NGF
U0126
NGF+U0126
100 nm
B
SK-MEL-28-TrkA
no Dox
Vehicle
NGF
U0126
NGF+U0126
G-361-TrkA
no Dox
Vehicle
NGF
U0126
NGF+U0126
SK-MEL-28-E
no Dox
Vehicle
NGF
U0126
NGF+U0126
G-361-E
no Dox
Vehicle
NGF
U0126
NGF+U0126
100 nm
